# The Heliosphere and Local Interstellar Medium from Neutral Atom Observations at Energies Below 10 keV

**DOI:** 10.1007/s11214-022-00901-7

**Published:** 2022-05-23

**Authors:** André Galli, Igor I. Baliukin, Maciej Bzowski, Vladislav V. Izmodenov, Marc Kornbleuth, Harald Kucharek, Eberhard Möbius, Merav Opher, Dan Reisenfeld, Nathan A. Schwadron, Paweł Swaczyna

**Affiliations:** 1 Physics Institute, University of Bern, Bern, Switzerland; 2 Space Research Institute of Russian Academy of Sciences, Moscow, Russia; 3 Moscow Center for Fundamental and Applied Mathematics, Lomonosov Moscow State University, Moscow, Russia; 4 Space Research Centre, Polish Academy of Sciences, Warsaw, Poland; 5 Boston University, Boston, USA; 6 University of New Hampshire, Durham, USA; 7 Los Alamos National Laboratory, Los Alamos, USA; 8 Department of Astrophysical Sciences, Princeton University, Princeton, NJ USA

**Keywords:** Interstellar medium, Heliosphere, Solar wind

## Abstract

As the heliosphere moves through the surrounding interstellar medium, a fraction of the interstellar neutral helium, hydrogen, and heavier species crossing the heliopause make it to the inner heliosphere as neutral atoms with energies ranging from few eV to several hundred eV. In addition, energetic neutral hydrogen atoms originating from solar wind protons and from pick-up ions are created through charge-exchange with interstellar atoms.

This review summarizes all observations of heliospheric energetic neutral atoms and interstellar neutrals at energies below 10 keV. Most of these data were acquired with the Interstellar Boundary Explorer launched in 2008. Among many other IBEX breakthroughs, it provided the first ever all-sky maps of energetic neutral atoms from the heliosphere and enabled the science community to measure in-situ interstellar neutral hydrogen, oxygen, and neon for the first time.

These observations have revolutionized and keep challenging our understanding of the heliosphere shaped by the combined forces of the local interstellar flow, the local interstellar magnetic field, and the time-dependent solar wind.

## Introduction

This paper gives an overview of the so far available observations in space of neutral atoms over the energy range from 10 eV to roughly 6 keV and their implications to heliospheric science. This overview includes both interstellar neutral (ISN) atoms (species-dependent energies from 10 eV to 0.8 keV for ram observations in Earth orbit), and Energetic Neutral Atoms (ENA) from the heliosheath (the plasma region between the solar wind termination shock and the heliopause) and from the perturbed interstellar medium outside the heliopause.

We adhere to the following nomenclature in this paper: “Heliosheath” is used instead of “Inner Heliosheath”; the region of perturbed interstellar medium just beyond the heliopause cannot be properly named “Outer Heliosheath” because it is currently unknown if there is a global bow shock around the heliopause. For the plasma regions outside the heliopause, “Very Local Interstellar Medium” (VLISM) is used to describe all regions surrounding the heliopause that are notably affected by the presence of the heliosphere (see Zank 2015, e.g., who defined the VLISM as “the region of the ISM surrounding the Sun that is modified by the deposition of heliospheric material”). The terms “LISM” or “pristine VLISM” are reserved for the medium at heliocentric distances where the presence of the heliosphere is irrelevant.

An ENA is formed when a fast ion (in this paper usually a proton with an energy between 10 eV and 6 keV) exchanges its charge with an ambient neutral atom (in this paper usually neutral hydrogen). The ENA then leaves its place of origin with almost the same momentum as the parent ion on a straight trajectory and can reach an ENA instrument far away. The detected ENA intensity can be written as the line-of-sight integral over the local proton intensity $j_{p}$ times the neutral hydrogen density $n_{H}$ times the charge-exchange cross-section $\sigma _{p,H}$:

1 $$ j_{\text{{ENA}}} = \int _{\text{inst}}^{\infty}dr \, j_{p}(r) \, n_{H}(r) \, \sigma _{p,H}. $$

ENA imaging thus allows for remotely sensing plasma regions. The interpretation of ENA measurements leads to an inversion problem because the measured ENA intensity is a line of sight integral over local ion intensities (possibly belonging to different populations) times the local neutral density times the energy-dependent charge-exchange cross-section. Thus, heliosphere models including ions and neutrals are often required to quantitatively interpret ENA measurements.

This paper complements the paper by Dialynas et al. (2022) in the same topical collection that discusses observations of high-energy ENAs (above 6 keV) and their implications for heliospheric science. Further articles directly related to this paper are: Theory and modeling of pick-up ions (PUI) and ENAs by Sokół et al. (2022), observations of interstellar PUI by Zirnstein et al. (2022), in-situ observations of the outer heliosphere by Richardson et al. (2022), heliosphere models by Kleimann et al. (2022), the observation of backscattered Ly-$\alpha $ emissions by Baliukin et al. (2022) and the article on the Local Interstellar Medium by Linsky et al. (2022).

This paper is structured as follows: The Interstellar Boundary Explorer and its instruments are briefly introduced, along with the few other space missions that have enabled the direct detection of interstellar neutrals and ENAs (Sect. 2). This is followed by the summary of the ENA and ISN observations (Sect. 3), grouped into the globally distributed flux of ENAs, the ENA Ribbon, the ISN He, ISN H, and ISN heavy species (O, Ne, and others). In Sect. 4, the implications of these observations for our understanding of the heliosphere and its local neighborhood are discussed. The paper is concluded by a summary and an outlook for upcoming space missions to measure neutrals from the heliosphere and the interstellar medium (Sect. 5).

## Space Missions Sampling Neutral Atoms

### GAS on Ulysses

GAS was the interstellar neutral-gas experiment on Ulysses (Witte et al. 1992). The GAS instrument detected ISN helium by their release of ions from a freshly deposited lithium-fluoride layer. The deep-space mission Ulysses (Wenzel et al. 1992) by ESA and NASA was active from 1990 until 2009, providing among many other achievements the first-ever measurements of regions above the Sun’s poles. Ulysses performed measurements during the cruise phase from launch to the gravity assist maneuver at Jupiter in 1992. Subsequently, GAS observed the ISN flow during each fast latitude scan near perihelion on the almost polar Ulysses orbit, maximizing the velocity relative to the interstellar gas. Before the end of the mission in 2009, the spacecraft completed three revolutions around the Sun.

Ulysses was a spin-stabilized spacecraft with the spin axis directed towards the Earth. The GAS instrument was mounted on a stepping platform that allowed to adjust the viewing direction relative to the spin axis of the spacecraft. This configuration allowed taking images of selected portions of the sky. In the ISN He observation mode, a selected sky region was scanned during one or two days, building complete images of the ISN He beam.

The measurement technique was based on counting events due to $\text{Li}^{+}$ ions impacting a channeltron detector. These ions were released from a LiF-covered lead-glass surface by impacting particles (sputtering) and photons. Because the sputtering process required a sufficiently high energy, the ISN flow observations were restricted to the cruise phase to Jupiter and to the fast latitude scans before and after perihelion of the Ulysses orbit. On the other hand, the instrument was sensitive both to neutral atoms and to EUV photons, with no intrinsic method of discriminating between interstellar He atoms and the unwanted background of EUV and X-ray photons. The ISN observation phases were therefore restricted to times when a strong ISN He flow was expected. The bright EUV stars, visible in these data as point sources, helped during the analysis to verify the actual pointing of the instrument during observation.

### ASPERA on Mars Express and Venus Express

Mars Express and Venus Express are two almost identical spacecraft of the European Space Agency (ESA). Mars Express was launched in 2003 and entered into Mars orbit in December of the same year (Chicarro et al. 2004); Venus Express was launched in 2005 and entered into Venus orbit in 2006 (Svedhem et al. 2009). As of 2022, Mars Express is still operational, whereas Venus Express reached its end of life in 2014.

The instrument suites ASPERA-3 (Barabash et al. 2006) and ASPERA-4 (Barabash et al. 2007) consist of an electron spectrometer, an ion mass analyzer to measure planetary and solar wind ions, and two ENA imagers. In this study, only the Neutral Particle Detector (NPD) is considered. NPD measured ENAs between 0.3 and 10 keV with Microchannel Plates (MCPs) registering the start and stop counts of secondary electrons triggered by an incoming ENA. The main objective of NPD was to measure ENAs from the Mars and Venus atmosphere and their plasma environments. However, both instruments could be operated on a few occasions during the cruise phase to Mars and Venus in 2003 and 2005/2006, respectively. These observations benefited from low background levels in interplanetary space, allowing for the observation of non-planetary ENAs far away from Earth and its magnetosphere.

### The Interstellar Boundary Explorer Mission (IBEX)

**Fig. 1:**
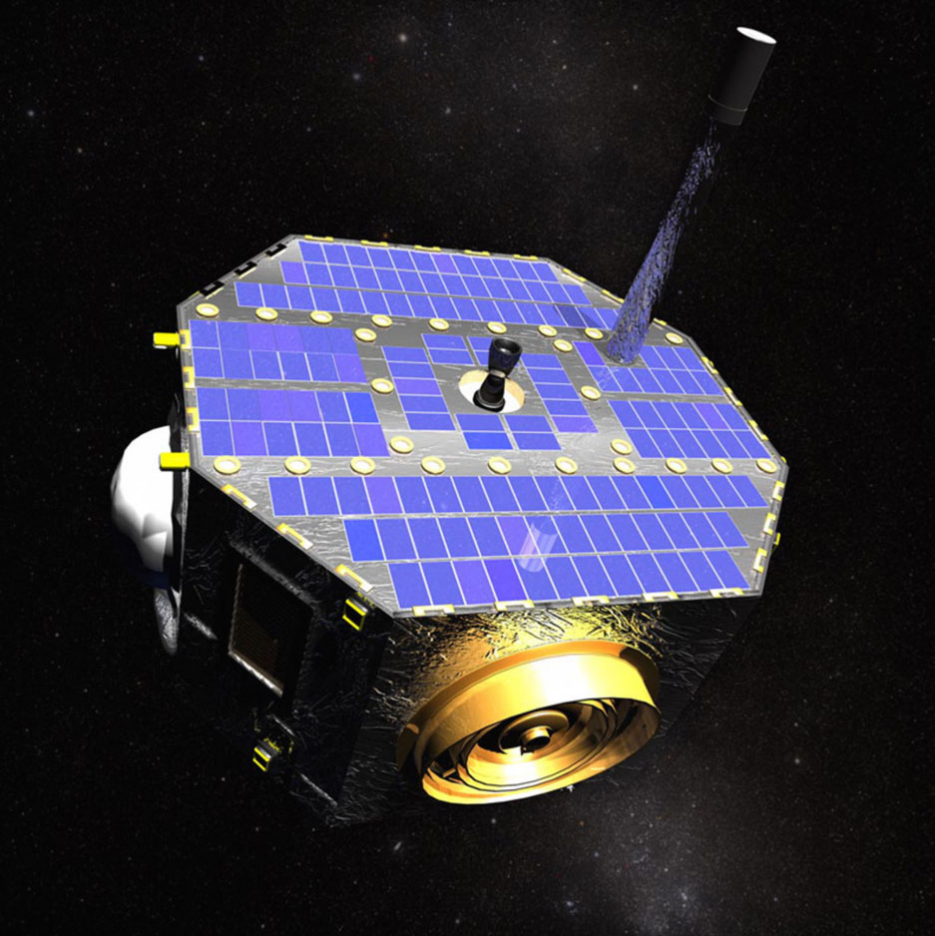
Artist’s rendition of the IBEX satellite, taken from McComas et al. (2009b). IBEX spins around the sun-pointing axis, the sun-oriented plate is covered with solar cells. The instrument on the bottom is IBEX-Lo, IBEX-Hi (not shown here) points to the opposite direction

IBEX is a small explorer mission of NASA (McComas et al. 2009b) placed in an elongated elliptical Earth orbit. The satellite is less than 1 m in diameter and carries two scientific instruments: The ENA-imagers IBEX-Hi and IBEX-Lo (see artist’s impression in Fig. 1).

IBEX was launched in 2008 and is successfully operating ever since in Earth orbit. Its science objective is to “discover the global interaction between the solar wind and interstellar medium” (McComas et al. 2009b). To achieve that objective, IBEX-Lo and IBEX-Hi measure ENAs from the heliosheath and the inflow of ISN from beyond the heliopause.

IBEX-Hi measures hydrogen ENAs between 0.5 and 6 keV. Incoming ENAs are converted to positive ions when passing through thin carbon foils. These ions are accelerated, guided through an electrostatic analyzer, and registered in the detector subsystem consisting of three chambers separated by carbon foils. Secondary electrons generated by ions passing through the carbon foils are registered by CEMs attached to each chamber (Funsten et al. 2009). IBEX-Lo uses conversion surfaces covered with diamond-like carbon to convert the incoming atoms into negative ions via charge-exchange and surface sputtering passing an electrostatic analyzer before post-accelerating the ions and detecting them in a triple time-of-flight (TOF) system with MCP detectors (Fuselier et al. 2009b). He and Ne atoms create only meta-stable negative ions upon charge-exchange (Wurz et al. 2008), which means the ionization efficiency of He and Ne atoms from the ISN flow is very low. Therefore, IBEX-Lo detects ISN He atoms mostly through $\text{H}^{-}$ and ISN Ne through $\text{H}^{-}$, $\text{O}^{-}$, and $\text{C}^{-}$ sputtered off the conversion surface and a thin water layer, which exists on top of the conversion surface (Möbius et al. 2009a; Bochsler et al. 2012). The energy spectrum of the sputtered species is much broader than the spectrum of the incoming atoms, filling all energy steps below the energy of the incoming ISN atoms (Möbius et al. 2012). IBEX-Lo covers an energy range of 2.5 keV down to 10 eV. At overlapping energies, IBEX-Hi offers a better signal-to-noise ratio than IBEX-Lo. On the other hand, the TOF information obtained in IBEX-Lo allows us to distinguish between different neutral species. This is important for measuring ISN flow, which does not only contain hydrogen, but also helium, oxygen, and other species.

## Observations of Energetic Neutral Atoms and Interstellar Neutrals

Observations of neutral atoms can be grouped into several types of ENA and ISN sources. When the first images of heliospheric ENAs were obtained with IBEX-Hi and IBEX-Lo late in 2009, it was soon realized that the ENA emissions could be characterized as a rather uniformly distributed ENA emission across the sky plus a conspicuous ribbon of brighter ENA emissions whose very existence came as a surprise. This ENA Ribbon turned out to be a $10^{\circ}\text{--}30^{\circ}$ wide feature of enhanced ENA fluxes superimposed on the globally distributed ENA flux. It is most prominent around solar wind energies and its shape and intensity follow the evolution of the solar wind over the solar cycle.

In the following years of analysis, it proved fruitful to separate the two ENA emissions to study their characteristics such as temporal evolution and energy spectrum and link them to potential ENA sources inside or outside the heliosphere. We will follow this approach by discussing first the globally distributed ENA signal in Sect. 3.1, followed by the IBEX ENA Ribbon in Sect. 3.2.

The neutral atoms in the VLISM, such as H, He, O, and Ne, have a large mean-free path for charge exchange, which is comparable (for H and O) with or larger (for He and Ne) than the characteristic size of the heliosphere (e.g. Izmodenov 2001). Therefore, these ISN atoms can penetrate the heliosphere due to the relative motion of the Sun and VLISM and can be measured in many cases as PUIs (Zirnstein et al. 2022) or even directly sampled in the inner solar system at energies between 10 eV and 660 eV. The distributions of these atoms carry information about their abundance in the VLISM and also about the filtration processes in the heliospheric boundary. The ISN elements observed so far are discussed separately in this paper: helium is the dominant species in the inner heliosphere, hydrogen is the second most abundant species in the inner heliosphere but is strongly affected by solar activity, and oxygen and neon are the most common heavy ISN species in the inner heliosphere.

### The Globally Distributed Signal of Energetic Neutral Atoms from the Heliosheath

**Fig. 2:**
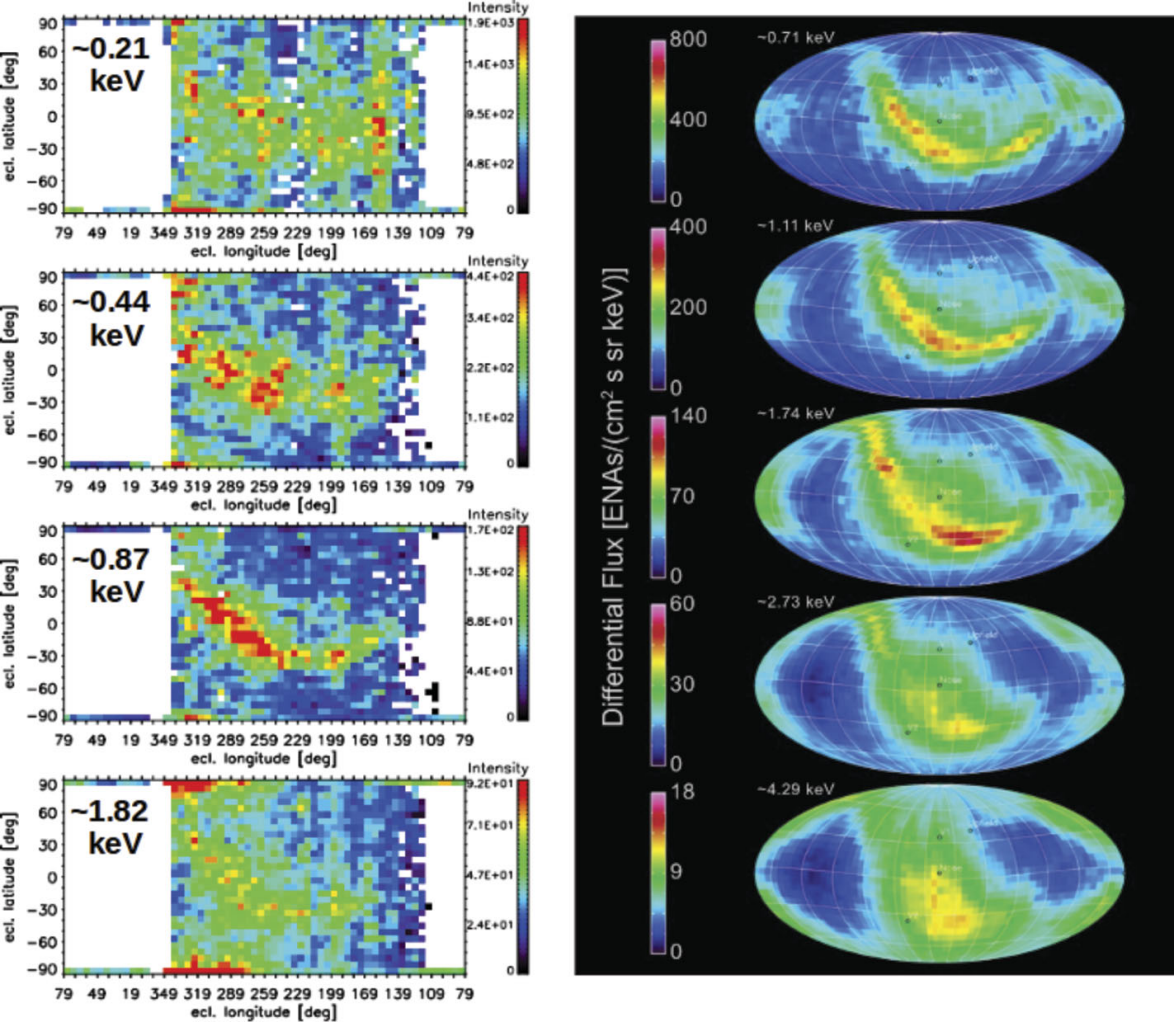
ENA intensity maps obtained with IBEX-Lo (left panel, figure taken from Galli et al. 2022) and IBEX-Hi (right panel, figure taken from McComas et al. 2020) in units of $\text{cm}^{-2}\,\text{sr}^{-1}\,\text{s}^{-1}\,\text{keV}^{-1}$. The maps are averaged over all 11 years of ENA ram observations and correspond to the spacecraft reference frame

To give the reader an overview of IBEX-Hi and IBEX-Lo ENA observations, we first show in Fig. 2 composite ENA images averaged over the first complete solar cycle covered with IBEX from 2009–2019. The IBEX-Lo maps are taken from Galli et al. (2022) (left panel), the IBEX-Hi maps were published in McComas et al. (2020) (right panel).

The term “Globally distributed ENA flux” (GDF) was introduced by Schwadron et al. (2014b) who refined an approach from Schwadron et al. (2011) to separate the Ribbon ENAs from the GDF to study their characteristics. This way, GDF refers to all observed heliospheric ENA emissions after subtraction of the Ribbon.

**Fig. 3:**
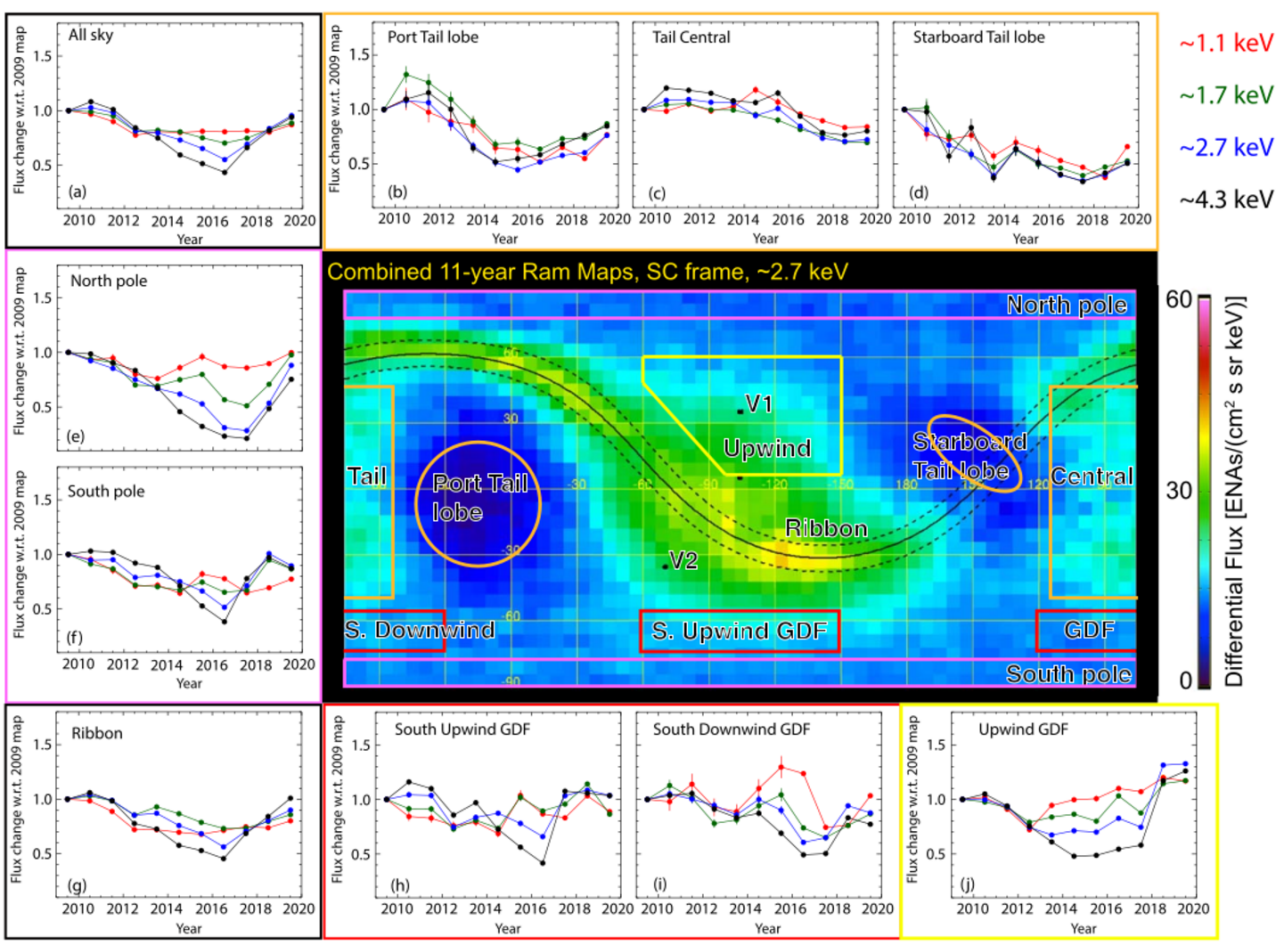
Temporal variability of ENAs measured with IBEX-Hi: Combined 11-year ram map at 2.7 keV with nine sky regions identified for temporal analysis. The surrounding panels show the year-by-year temporal variations for the average ENA fluxes normalized to the 2009 fluxes for each region at all energies, along with an average over the whole sky (upper left). The energy passbands are color coded (upper left). Figures taken from McComas et al. (2020)

We clearly observe solar cycle effects on the GDF in the IBEX data. This evolution is illustrated in Fig. 3 from McComas et al. (2020), which also gives a good summary of the different ENA sky regions in the IBEX energy range: The upwind or helionose region lies close to the IBEX Ribbon, Voyager 1 and Voyager 2 crossed the heliopause on either side of the Ribbon, the downwind direction is designated as Central Tail region. The GDF is rather uniform (the bluish pixels to the North and South in Fig. 3), but two large lobes of depleted ENA intensity on either side of the central tail region can be identified. The basic temporal evolution of the all sky GDF and of most subregions exhibits a negative correlation of observed ENA intensities at 1 au with solar activity (as expected from ionization rates changing with solar activity Sokół et al. 2020).

**Fig. 4:**
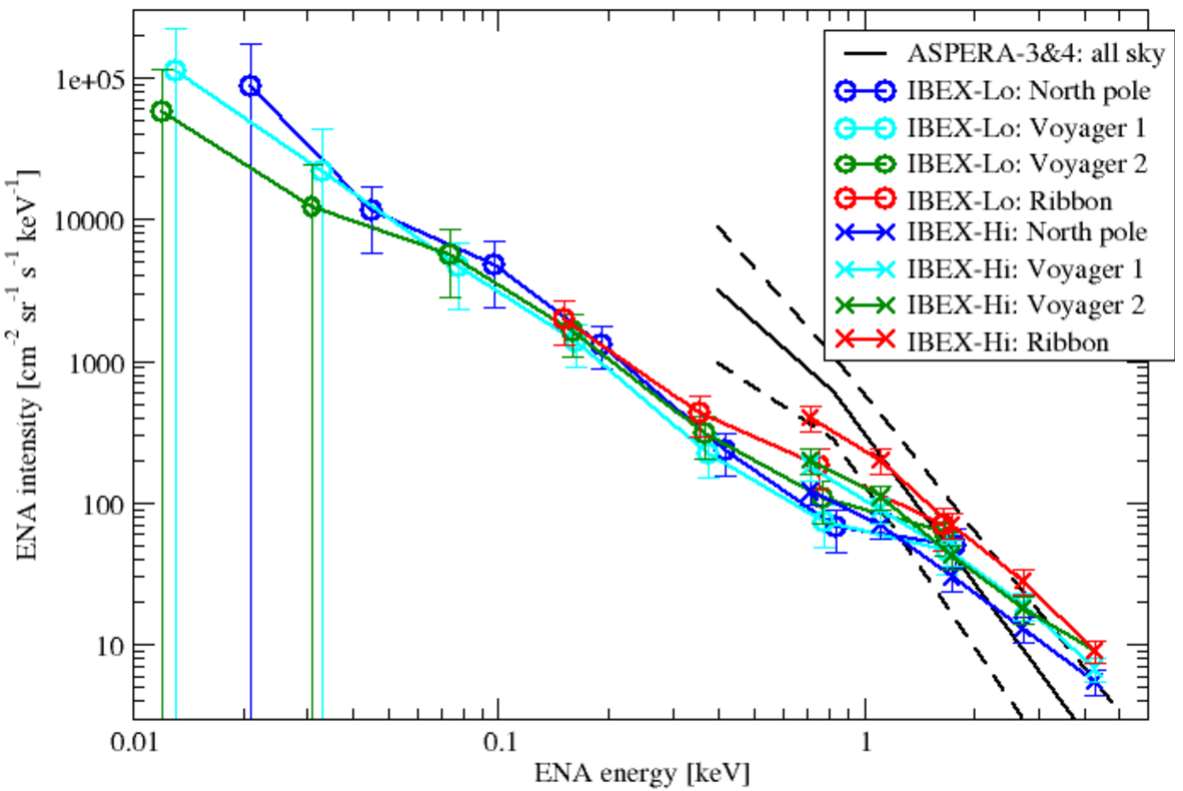
ENA energy spectra in inertial reference frame at 100 au for 4 different viewing directions (colored labels) for the 11-year average ENA intensities 2009–2019, measured with IBEX-Lo (Galli et al. 2022), IBEX-Hi (McComas et al. 2020) and the all-sky ENA spectrum observed with ASPERA-3&4 in 2003 and 2005 (Galli et al. 2013)

The ENA energy spectrum of the GDF and the Ribbon are shown in Fig. 4, based on measurements with IBEX-Lo (Galli et al. 2022), IBEX-Hi (McComas et al. 2020), and ASPERA-3&4 in 2003 and 2005 (Galli et al. 2013) (all sky only, as the data were too sparse to differentiate between sky regions). The IBEX observations in this plot are averages over the 11 years from 2009–2019. The error bars represent absolute uncertainties (Fuselier et al. 2014; Galli et al. 2014).

The energy spectra in Fig. 4 confirm that the GDF is an apt description of this ENA emission, as the intensities look rather uniform across the sky (compare blue, cyan and green curves) with the exception of the Ribbon regions around solar wind energies (red curves). The GDF spectra at energies above solar wind energies (1 keV) measured with IBEX-Hi can be characterized by a power law with a spectral index of roughly −2.0. It is steeper ($\sim -2.5$) in the ecliptic plane and flatter ($\sim -1.5$) in polar regions (Funsten et al. 2009; Desai et al. 2016, 2019; McComas et al. 2020). Between 1 keV and 100 eV, the ENA energy spectra look similar for all sky regions studied (with the exception of the IBEX Ribbon) with a spectral index of $-1.8\pm 0.2$ also for regions close to the ecliptic plane (see Fig. 4). The ENA energy spectra flatten significantly to a spectral index of $>-1$ below 100 eV ENA energy. Below 50 eV, the derived ENA intensities are difficult to constrain against the background sources (Fuselier et al. 2014; Galli et al. 2014) and the derived energy spectra entries below that energy have a large error bar (see error bars in Fig. 4). For a heliospheric ENA energy spectrum spanning all observed energy ranges up to 55 keV, the reader is referred to Dialynas et al. (2020) and to the accompanying reviews by Dialynas et al. (2022) and Kleimann et al. (2022).

From the beginnings of the IBEX mission, interpretations of the GDF (Fuselier et al. 2009a; McComas et al. 2009a; Hsieh et al. 2010; Schwadron et al. 2011) were based on the assumption that the GDF ENAs likely originate from charge exchange between interstellar neutrals and plasma in the heliosheath. One alternative origin would be heated protons in the VLISM outside the heliopause. Any feasible mechanism must account for the observational fact that the GDF is also observed from the downwind heliosphere direction. For the GDF spectrum around solar wind energy (above 0.5 keV), PUIs in the heliosheath may suffice (Galli et al. 2014; Zirnstein et al. 2014 but also see Fuselier et al. 2021). The latitudinal ordering of ENA energy spectra around solar wind energy probably reflects the different solar wind speed for equatorial and polar heliodirections (Desai et al. 2015). The case remains less clear for lower ENA energies: Based on model comparisons by Zirnstein et al. (2014), Galli et al. (2014) found that a large fraction of the observed GDF energy spectrum below 0.5 keV could be reproduced by PUIs from the heliosheath. Zirnstein et al. (2018c) derived that solar wind protons and PUIs propagating down the heliotail and charge-exchanging with neutral hydrogen atoms as source terms can reproduce the observed GDF spectrum from the downwind hemisphere to the lowest observed energies if stochastic acceleration of PUI via turbulence is included. Otherwise their model underestimated the measured ENA intensities by a factor of 2–3 over all energies. Zirnstein et al. (2018c) also predicted that the ENA spectrum should flatten but not roll over below 0.1 keV, because low energy PUIs are continually injected into the plasma from charge-exchange with neutral hydrogen propagating into the heliotail. Mostafavi et al. (2019) found that their model can reproduce the ENA intensities observed with IBEX-Hi around solar wind energies only if strong shocks in the heliosheath are included. Currently, most model predictions tend to underestimate the observed GDF ENA intensities over the entire energy range accessible with ENA observations from roughly 50 eV to 55 keV (also see Dialynas et al. 2022; Giacalone et al. 2022; Kleimann et al. 2022).

Expanding ENA observations with IBEX over a full solar cycle has increased the discrepancy between the observed ENA intensities and model predictions: the ENA intensities below 0.5 keV measured after 2012 tend to be higher compared to the early years 2009–2012 (compare e.g. energy spectra from Galli et al. 2016 and Galli et al. 2022 for the Voyager 2 direction). A more recent model-observation comparison by Fuselier et al. (2021) based on IBEX observations from 2009–2018 found that heliosheath PUIs can only account for 10% at most of the observed ENAs from the Voyager 2 direction for any energy below 2 keV. This implies that the majority of GDF ENAs does not originate from protons in the heliosheath but rather from protons in the VLISM (Fuselier et al. 2021). One candidate are secondary ENAs from PUI (Chalov et al. 2010; Heerikhuisen et al. 2010; Zirnstein et al. 2016b) outside the heliopause in a process similar to the one presumed for the Ribbon ENAs (see Sect. 3.2). The discrepancy between ENA intensities observed with IBEX and model predictions was only a factor of 2 in the analysis by Kornbleuth et al. (2021), but the authors confirmed the underestimation of the GDF for two different heliosphere models and also found that the discrepancy increases at the lowest observed IBEX energies. The observed discrepancy could be explained by the low density of ISN hydrogen assumed in the models compared to the most recent estimates of the density inferred from New Horizons/SWAP PUI observations (Swaczyna et al. 2020). A higher ISN hydrogen density implies a higher density of PUIs and also a higher neutralization rate in the heliosheath.

### The IBEX ENA Ribbon

#### The Origin of the IBEX Ribbon

**Fig. 5:**
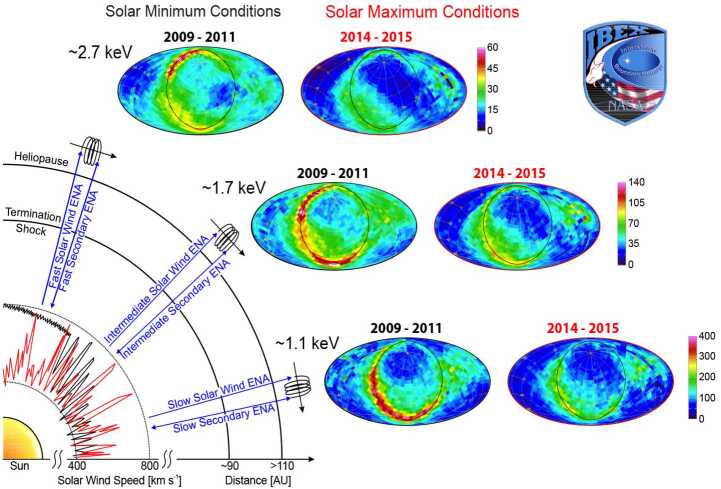
The IBEX Ribbon is naturally ordered by the solar wind. Near solar minimum (2009–2011), IBEX observed higher energy ENAs emitted preferentially from higher latitudes and lower energy ENAs from lower latitudes. This high versus low latitude ordering of Ribbon ENAs reflects the latitudinal ordering of solar wind in a solar minimum configuration with fast solar wind emitted from high latitude coronal holes and slower wind emitted from the streamer belt region. Nearer solar maximum, when correcting for transit time effects (2014–2015), IBEX observes a distribution of ENAs similar at high and low latitudes, reflecting the solar wind distribution near solar maximum. From McComas et al. (2012, 2017)

IBEX discovered the completely unanticipated Ribbon emission from the first observations in 2009 (McComas et al. 2009a). The Ribbon stretches as a $20^{\circ}$-wide swath of the sky (Fuselier et al. 2009a) along a circle extending $\sim75^{\circ}$ from a Ribbon center located at ecliptic $(\lambda ,\beta )=(219.2^{\circ}, 39.9^{\circ})$ (Funsten et al. 2013). First, its origin was an enigma and thus attracted numerous origin theories (McComas et al. 2009a, 2018b; Chalov et al. 2010; Gamayunov et al. 2010, 2017; Grzedzielski et al. 2010; Heerikhuisen et al. 2010; Fahr et al. 2011; Siewert et al. 2012, 2013; Kivelson and Jia 2013; Kucharek et al. 2013; Schwadron and McComas 2013; Fichtner et al. 2014; Isenberg 2014; Giacalone and Jokipii 2015; Schwadron et al. 2018). ENA fluxes from the Ribbon in the IBEX energy range show a clear connection with the latitudinal ordering of the solar wind (Fig. 5), thus supporting a secondary ENA source beyond the heliopause. Significant progress has been made in understanding how IBEX and Voyager observations constrain Ribbon origin theories (Gamayunov et al. 2017, 2019; Zirnstein et al. 2018a, 2019a, 2020a). Active debates continue regarding the physics involved in the creation of the Ribbon.

Initial observations of the Ribbon (McComas et al. 2009a) revealed that it is likely ordered by the local interstellar magnetic field (LISMF) (Schwadron et al. 2009; Funsten et al. 2013). In fact, the LISMF direction deduced from the Ribbon correlates well with large-scale anisotropies of TeV GCRs (Schwadron et al. 2014a) and is consistent with the large-scale field deduced from polarized starlight (Schwadron et al. 2014a; Frisch et al. 2015). The LISMF properties remain controversial. Some studies conclude that the termination shock asymmetries are explained with a stronger LISMF less-inclined to the ecliptic plane (Izmodenov et al. 2009; Opher et al. 2009). Other studies conclude that the “Belt”, a broad ENA emission observed by Cassini/INCA (Krimigis et al. 2009; Dialynas et al. 2022) that follows a different symmetry axis than the Ribbon, requires a vastly different LISMF.

Since IBEX observes each direction of the sky twice per year, the Ribbon’s apparent position on the celestial sphere is shifted. Using 5 years of data, Swaczyna et al. (2016a) found that the Ribbon shifts by a parallax angle of $0.41^{\circ}\pm 0.15^{\circ}$, corresponding to a source $140^{+84}_{-38}~\text{au}$ from the Sun, consistent with sources located outside the heliopause.

**Fig. 6:**
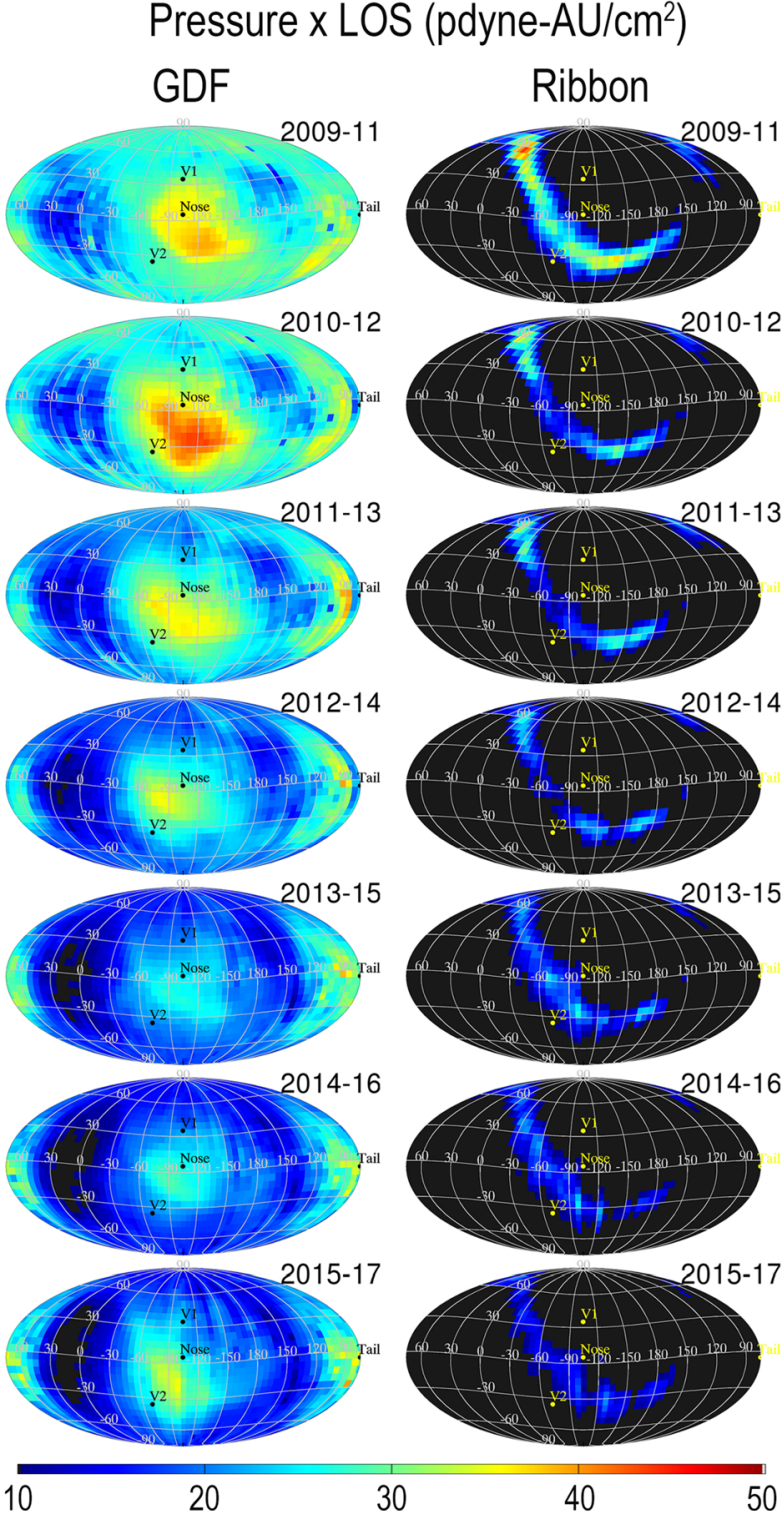
LOS-integrated pressure maps of GDF and Ribbon flux observed by IBEX. The time variation of the Ribbon flux lags the GDF, in support of a secondary ENA source for the Ribbon beyond the heliopause. From Schwadron et al. (2018)

The latest IBEX observations show a disparity in the evolution of the Ribbon and the surrounding GDF (Schwadron et al. 2018; McComas et al. 2017; Swaczyna et al. 2021a). Over the first 5 years of observations, the GDF was generally dimming over time, while parts of the Ribbon showed a leveling or slight increase (McComas et al. 2014a). However, a recent analysis of 9 years of IBEX data show that the LOS-integrated ion pressure in the Ribbon has been steadily decreasing over the course of the mission, whereas since 2015, the GDF pressure at the nose has been increasing (Fig. 6). This behavior is consistent with the recovery in solar wind dynamic pressure now being reflected in the GDF with a longer delay for the Ribbon, adding support to a secondary ENA Ribbon source beyond the heliopause (Schwadron et al. 2018).

#### The Source of the IBEX Ribbon

A large array of possible sources for the IBEX Ribbon have been proposed (McComas et al. 2009a, 2014b; Swaczyna et al. 2016a). Continued IBEX observations have made it evident that one scenario involving “secondary ENAs” is consistent with observations of the Ribbon over an entire 11-year solar cycle (McComas et al. 2020). The Ribbon source from secondary ENAs begins with neutralized solar wind protons that form neutral solar wind (primary ENAs) moving essentially radially out beyond the heliopause. There is a special locus of directions where the outward neutral (or secondary) solar wind is roughly perpendicular to the local interstellar magnetic field that drapes around the heliosphere. Along this locus of directions the dot product of the draped local interstellar magnetic field $\mathbf {B}$ with the radial direction $\mathbf {r}$ from the Sun is roughly zero, $\mathbf {B}\cdot \mathbf {r}\approx 0$. Charge-exchange with interstellar $\text{H}^{+}$ ionizes the neutral solar wind H atoms and thereby creates pick-up protons that initially gyrate about the local interstellar magnetic field to form ring-beam distribution. Next, these protons that initially gyrate almost perpendicular to the magnetic field undergo a final charge-exchange to form secondary neutral H atoms. If this last charge-exchange occurs at the correct phase of the gyration, the emerging ENAs travel back into the heliosphere to the IBEX satellite where they are detected (McComas et al. 2009a).

The major challenge for the secondary ENA model of the Ribbon has been the need of a long-term (a few years) stability of the PUIs in the ring distribution despite the susceptibility of such a distribution to instabilities and subsequent scattering (Florinski et al. 2010). Therefore, the mechanism has been modeled in detail by a growing list of studies. There are two major variants of secondary ENA models for the IBEX Ribbon:

1. If the initial ring-beam formed beyond the heliopause can maintain its stability over years, then particles from this ring-beam distribution build up sufficient density to explain the fluxes of the observed from the IBEX Ribbon (Chalov et al. 2010; Heerikhuisen et al. 2010; Möbius et al. 2013; Zirnstein et al. 2016a, 2018a). Several mechanisms have been proposed that could substantially reduce the growth of instabilities (Liu et al. 2012; Summerlin et al. 2014; Florinski et al. 2016).
2. If protons created from the neutral solar wind (and other neutral sources) are retained within a spatial region close to the surface where $\mathbf {B}\cdot \mathbf {r}\approx 0$, then the Ribbon reflects the spatial region where these protons are retained (Schwadron and McComas 2013, 2019a, 2021; Isenberg 2014; Giacalone and Jokipii 2015). Multiple mechanisms may act to retain ions within the Ribbon. The initial ring-beam distribution likely excites ion-cyclotron waves that rapidly scatter ions near the $\mathbf {B}\cdot \mathbf {r}\approx0$ surface to retain protons (Schwadron and McComas 2013, 2019a; Isenberg 2014). Another possibility is that ions are trapped due to reflection by amplitude variations of the magnetic field (Giacalone and Jokipii 2015).

The secondary ENA mechanism can explain the source of Ribbon ENAs observed at solar wind energies ($\sim0.5\text{--}3~\text{keV}$), but the Ribbon is observed down to 0.2 keV (Fuselier et al. 2009a, 2018; Schwadron et al. 2014b) and up to at least 6 keV, where it may merge with or transition into the Cassini/INCA ENA Belt (Schwadron et al. 2014b; Dialynas et al. 2022). It has been discovered that a likely source of Ribbon emissions outside the solar wind energy range are neutral atoms created by charge-exchange with PUIs inside the termination shock and suprathermal ions in the heliosheath, having energies that cover the full observed Ribbon energy range (Schwadron and McComas 2019a; Fuselier et al. 2018; Schwadron and McComas 2019b). These ENAs are not all directed radially outward like the neutral solar wind. This can explain why the Ribbon at high and low energies is broader than at solar wind energies and why the Ribbon center shifts at higher energies, evolving naturally into the Belt.

#### Testing Ribbon Hypotheses

A necessary test of each Ribbon hypothesis is for a model to reproduce key observables, such as the spatial geometry, spectral properties, and variability of the ENA flux over time. The correlation between ENA fluxes and solar wind variation over the solar cycle is emerging as a definitive test. With the data covering a full solar cycle, we can begin to eliminate potential Ribbon mechanisms. For example, the H-wave scenario (Fichtner et al. 2014; Sylla and Fichtner 2015) predicts the Ribbon is formed from charge-exchange in the heliosheath with a high density H-wave moving through space. This implies that the Ribbon’s evolution would vary similarly with the surrounding GDF and that the Ribbon’s celestial position will move in the sky. The Ribbon’s position and radius appear to be stable over time (Dayeh et al. 2019). Thus, the viability of the H-wave scenario is becoming unlikely. Moreover, the Ribbon’s delayed evolution compared to the GDF may eliminate a termination-shock-based source and the parallax results and the temporal variations strongly argue against a Ribbon source at the edge of the Local Interstellar Cloud (Grzedzielski et al. 2010).

It appears that the secondary ENA hypothesis is the most likely origin of the Ribbon (e.g. Zirnstein et al. 2015; McComas et al. 2017; Schwadron et al. 2018). Because it takes considerably longer for secondary ENAs from this mechanism to reflect changes in the solar wind compared to the GDF (Zirnstein et al. 2015), an important test is a comparison of the Ribbon’s evolution with the GDF. IBEX data (Schwadron et al. 2018) show a significant reduction in Ribbon flux over 9 years at all latitudes. The GDF in the noseward direction (Fig. 6) is strongly enhanced in recent years by a large increase in solar wind pressure in 2014. If the secondary ENA hypothesis is correct we expect the Ribbon, particularly in the noseward hemisphere, to brighten in the next few years (2021–2023), reflecting the neutral solar wind associated with the pressure pulse passing into the plasma regions around the heliopause.

**Fig. 7:**
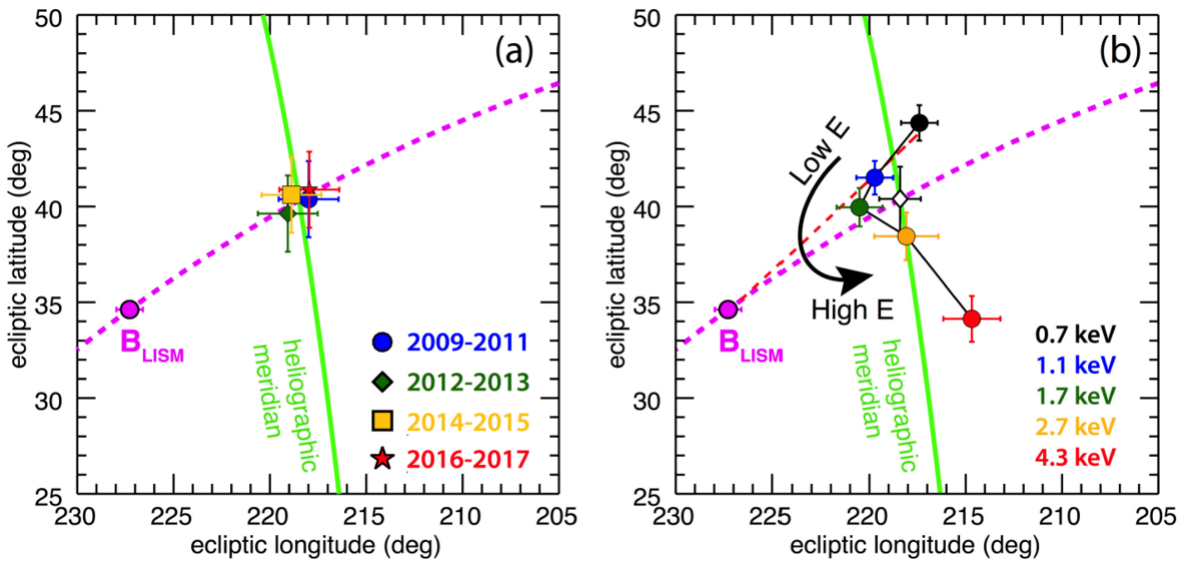
Left panel: Derived Ribbon centers for the five IBEX-Hi energy passbands during four different time periods, showing the intrinsic stability of the Ribbon centers over time. Right panel: Shift in the Ribbon centers as a function of energy. The magenta curve traces the B-V plane ($\mathbf {B}_{\text{LISM}}$ from Zirnstein et al. 2016b, and $\mathbf {V}$ from McComas et al. 2015), the green line traces the heliographic meridian, which passes through the solar poles. The pristine interstellar magnetic field direction (Zirnstein et al. 2016b) is shown in magenta. The red dashed line is a fit to the Ribbon centers below 1.7 keV and the pristine interstellar magnetic field direction. The open diamond symbol is the averaged Ribbon center location over all energies. From Dayeh et al. (2019)

The Ribbon geometry is another useful observable to test the secondary ENA mechanism. Models reveal that the observed dispersion of the Ribbon center as a function of ENA energy is due to the fast-slow solar wind structure (Swaczyna et al. 2016b), and under isotropic solar wind conditions the Ribbon center is nearly identical at all energies (Zirnstein et al. 2016a). Thus, as the latitudinal solar wind structure changes over the solar cycle, we expect the Ribbon centers to change at different energies. Curiously, after 9 years, the Ribbon centers have not shifted appreciably (Dayeh et al. 2019), see Fig. 7.

It has long been known that the Ribbon’s transverse shape is approximately $20^{\circ}$ wide (Fuselier et al. 2009a). Most studies assume it is Gaussian-shaped, although a follow-up analysis of the Ribbon geometry showed it is more likely a skewed-Gaussian (Funsten et al. 2013). Two recent studies that simulated the Ribbon including three different PUI transport effects, i.e., (1) spatial retention of ions with enhancement in ion density in the retention region, (2) strong isotropic scattering in pitch angle without enhanced ion density, and (3) weak pitch angle scattering where ions maintain their initial pitch angles, showed that the Ribbon’s peak changes shape for each effect (see Zirnstein et al. 2019b,a, 2021 and Sect. 6.2 in Fraternale et al. 2022). Under weak scattering, the Ribbon shape is nearly Gaussian. The spatial retention model produces a skewed-Gaussian shape, although skewed in the direction opposite from data (Funsten et al. 2013). The isotropic scattering model predicts a skewed-Gaussian in the opposite direction compared to the spatial retention method, similar to the data, but with a much lower flux and thus inconsistent with the data.

The two variants of Ribbon models (spatial retention of ions in the Ribbon source region, and the weak pitch-angle scattering model which maintains the original ion pitch angles) give rise to very different apparent parallax distances (Zirnstein et al. 2019b). The spatial retention model predicts a parallax consistent with the source location, but the weak scattering model results in a parallax suggesting source at $\sim1000~\text{au}$, well outside data uncertainties.

One model based on the secondary ENA mechanism hypothesizes that the Ribbon is formed by the interaction of PUIs with interstellar turbulence (Giacalone and Jokipii 2015), predicting the Ribbon is double-peaked, which may be related to the possible fine structure seen in IBEX observations (McComas et al. 2009a). However, recent modeling efforts show that the double-peaked Ribbon prediction is not realistic, but an artifact of model assumptions (Zirnstein et al. 2020a). The existence of Ribbon fine-structure has not yet been determined from observations. But modeling developments (Zirnstein et al. 2020a) still predict that the Ribbon can exhibit fine-structure in the presence of turbulence.

#### Connecting the Ribbon with Voyager Observations

In August 2012, the Voyager 1 (V1) spacecraft crossed the heliopause (Gurnett et al. 2013; Stone et al. 2013), and has been measuring the draped LISMF. Schwadron et al. (2015b) showed that V1 was measuring a field direction $>40^{\circ}$ from both the IBEX Ribbon center and the symmetry plane containing the LISMF and LISM flow. The temporal evolution of the LISMF observed on V1 appeared to approach the direction of the Ribbon center during quiet periods unperturbed by shocks, but evolves away from the Ribbon center during active periods (Schwadron and McComas 2017). This indicates that V1 remains in the draped LISMF in a dynamic region of space (Burlaga and Ness 2016).

V1 crossing the heliopause enabled sophisticated Ribbon modeling and comparative analyses with the Ribbon position, leading to an inferred magnitude ($\sim2.9~\upmu \text{G}$) and direction ($227^{\circ},35^{\circ}$) of the pristine LISMF far from the Sun (Zirnstein et al. 2016b; Grygorczuk et al. 2011). These results support the concept that V1 is observing the draped LISMF. A recent analysis of 9 years of IBEX data shows that the Ribbon center is stable over much of the solar cycle (Zirnstein et al. 2015). Zirnstein et al. (2020b) analyzed the time variation of the Ribbon over the solar cycle and compared it with the expected changes in the parent neutral solar wind.

The Voyager spacecraft have made invaluable measurements of the heliosheath and VLISM plasma. Connecting Voyager measurements and IBEX ENA observations is essential for interpreting the Ribbon’s origin. The Ribbon center is widely believed to indicate the general direction of the LISMF (Schwadron et al. 2009), and a precise value for the pristine LISMF magnitude and direction have been self-consistently inferred using analyses between observation and model (Zirnstein et al. 2016b). However, V1 observed surprisingly similar directions for the heliospheric magnetic field and LISMF directions before and after it crossed the heliopause. This is now substantiated by V2, which also sees similar field directions on both sides of the heliopause (Burlaga et al. 2019). V1 observations are supported by several models and explained through LISMF draping at the heliolatitude of V1 (Opher and Drake 2013; Borovikov and Pogorelov 2014; Grygorczuk et al. 2014; Opher et al. 2017). It remains to be seen if such models can also explain the V2 data. As V1 and V2 travel away from the heliopause, they should encounter the pristine LISMF far from the heliosphere. Linear extrapolation of the changing LISMF direction observed by V1 (Schwadron et al. 2015b) shows that the LISMF will eventually approach the Ribbon center, and thus the pristine LISMF direction. But, V1 is in a region of space dominated by solar wind disturbances propagating across the heliopause, with recent LISMF measurements confirming a draped field structure (Schwadron et al. 2015b; Burlaga and Ness 2016). An important test of the Ribbon center as the signature of the LISMF direction is that Voyager data should show the observed LISMF eventually approaching the Ribbon center. Validation of this idea would support the alignment of the Ribbon center with the LISMF, and the secondary ENA source of the Ribbon.

### Interstellar Helium

Interstellar helium atoms have been observed to date by the GAS experiment onboard Ulysses (Witte et al. 1992) and IBEX-Lo onboard IBEX (Fuselier et al. 2009b). The analysis of the GAS ISN He observations provided a de facto standard of the ISN flow vector and the temperature of the local interstellar medium. The analyses converged to the J2000 inflow direction given by longitude $255.4^{\circ}\pm 0.5^{\circ}$, latitude $5.2^{\circ}\pm 0.2^{\circ}$, speed $26.3 \pm 0.4~\mbox{km}\,\text{s}^{-1}$, and a temperature of $6300 \pm 340~\text{K}$ (Witte et al. 1996, 2004; Witte 2004) in general agreement with PUI and UV backscattering observations (Möbius et al. 2004). These results were obtained by fitting the ISN He flow and temperature to observations from individual days and subsequently averaging the results.

First analyses of the IBEX-Lo observations suggested that the inflow velocity and the temperature of ISN He were different from those found by Ulysses (Bzowski et al. 2012; Möbius et al. 2012), which raised the question if the inflow direction of interstellar gas might be changing with time (Frisch et al. 2013). Bzowski et al. (2014) analyzed the Ulysses observations using a technique similar to that applied to IBEX observations, fitting the velocity vector and the temperature to the entire data set. The separation of observation seasons allowed these authors to perform a study of the temporal stability of the inflow parameters. Results of this study basically supported the inflow velocity vector obtained by Witte (2004) (longitude $255.3^{\circ}$, latitude $6^{\circ}$, speed $26.0~\text{km}\,\text{s}^{-1}$) but the temperature was found to be higher, about 7500 K. No indication was found that these parameters changed with time.

An independent analysis by Wood et al. (2015) relied on the same data set as Bzowski et al. (2014) but applied a different analysis method. Like Bzowski et al. (2014), they found no evidence for a change in the inflow parameters with time. The three-season average J2000 parameters were found to be longitude $255.54^{\circ}\pm 0.19^{\circ}$, latitude $5.44^{\circ}\pm 0.24^{\circ}$, speed $26.08 \pm 0.21^{\circ}$, and temperature $7260 \pm 270~\text{K}$ (the latter again significantly higher than the value derived by Witte 2004).

IBEX uses a markedly different observational strategy compared with Ulysses. The IBEX-Lo boresight points perpendicular to the spacecraft spin axis, which is repointed every few days to approximately follow the Sun (McComas et al. 2009b, 2011). Since the bulk ISN He flow is within a couple of degrees from the ecliptic plane, the bulk ISN He is in the IBEX-Lo field of view twice a year. ISN He atoms are gravitationally attracted by the Sun, so their bulk speed at 1 au is $\sim50~\text{km}\,\text{s}^{-1}$. In February, when IBEX moves together with Earth into the ISN He flow, the He atom speed in the instrument frame reaches about $50 + 30 = 80~\text{km}\,\text{s}^{-1}$, while in October, when IBEX moves with the ISN flow, this speed drops to $50 - 30 = 20~\mbox{km}\,\text{s}^{-1}$. These speeds correspond to the energy range from $\sim8$ to $\sim130~\text{eV}$. Galli et al. (2015) showed that ISN He atoms are not observed in the fall season, likely because the energy threshold of the sputtering process for He atoms in IBEX-Lo is $\sim17~\text{eV}$ (Sokół et al. 2015). During the favorable conditions in February, on the other hand, the ISN He atom energy falls within the nominal range of IBEX-Lo energy bin 4. Due to the sputtering process involved, $\text{H}^{-}$ and $\text{O}^{-}$ ions sputtered by ISN He atoms off the conversion surface are observed in the three lowest energy bins with similar intensities and with substantially reduced intensity in energy bin 4. Swaczyna et al. (2018) showed that the analysis of these observations leads to the same properties of the ISN He flow. However, the intensity in higher energy steps is smaller at higher ecliptic latitudes since the atom energy decreases as the boresight moves away from the ecliptic plane.

**Fig. 8:**
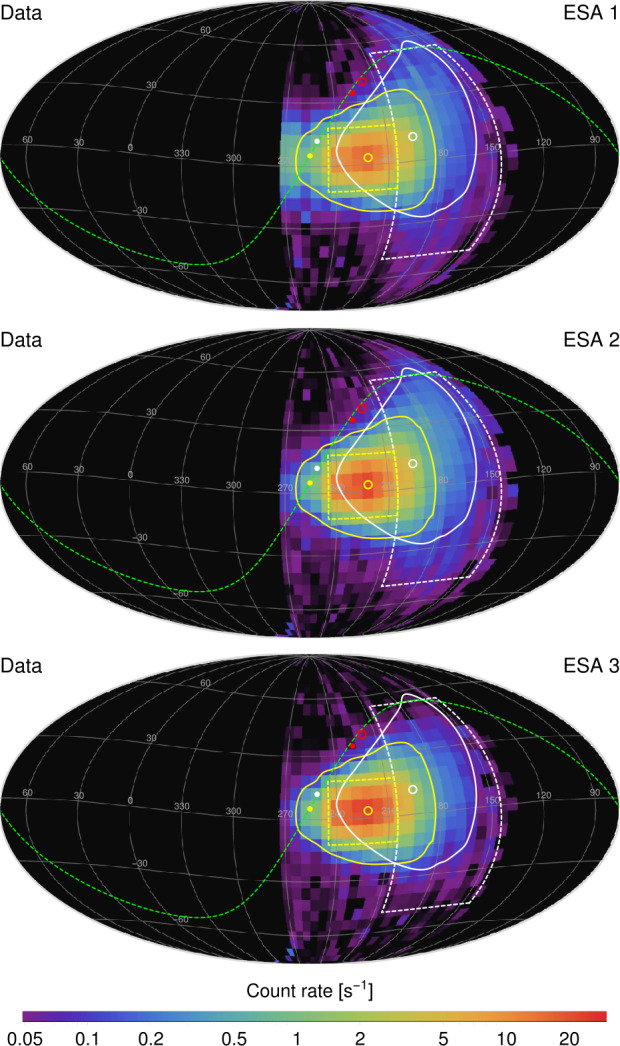
IBEX-Lo observed rates in energy steps 1, 2, and 3 (from top to bottom) during ISN observation seasons 2009–2015. Yellow and white lines encompass region of emission from the primary and secondary ISN helium, respectively. The peak positions (circles) are shifted from the inflow direction (points) due to solar gravity. Reproduced from Swaczyna et al. (2018)

The IBEX-Lo observations in the lowest ESA steps show a large dynamic range of count rates ranging from $\sim0.01~\text{s}^{-1}$ in the regions where only the ubiquitous background is observed (Galli et al. 2015) up to $\sim30~\text{s}^{-1}$ (Swaczyna et al. 2018) in the peak of the ISN He signal. Figure 8 shows the observed rates in the three lowest energy steps of IBEX-Lo. The angular extent of the observed structure in the ISN He signal is very narrow (Möbius et al. 2009b), and therefore, the precise pointing of the instrument is crucial for the interpretation of these observations.

A detailed uncertainty system covering the issues discussed above was developed by Swaczyna et al. (2015). In addition to the Poisson uncertainties, the system includes the throughput correction from the instrument to the electronics with its uncertainty, subtracts the average background, and accounts for uncertainties of the pointing direction. All components are included with appropriate correlations in the total covariance matrix.

The ISN He observations are used to find the properties of the pristine VLISM ahead of the heliosphere because it is the most abundant neutral species at 1 au (Sokół et al. 2019a) and it is much less modulated in the heliospheric boundaries compared to the ISN H (Ripken and Fahr 1983). Yet, there are two main components of the ISN He. The primary population (yellow ellipses in Fig. 8) originates from the pristine VLISM and was identified in the first observations from IBEX-Lo (Möbius et al. 2009b). The secondary population (white rectangles in Fig. 8) was initially identified as an additional population visible in the IBEX-Lo data earlier during the ISN seasons than the core ISN He (Kubiak et al. 2014, 2016; Park et al. 2016). Bzowski et al. (2017, 2019) showed that this population is created by charge exchange collisions between $\text{He}^{+}$ ions and ISN He atoms in the perturbed VLISM outside the heliopause, similarly to the secondary hydrogen. However, due to lower densities, this population is much less abundant than the primary population. Nevertheless, the secondary population contributes to the observed signal and causes broadening of the primary ISN He peak, which may affect the temperature and primary ISN flow direction determined from the IBEX-Lo observations (Möbius et al. 2015). Theoretical efforts to better understand secondary ISN populations in the heliosphere are presented in more detail in the review by Sokół et al. (2022) (Sect. 8.3).

He atoms are attracted in the Sun’s gravitational field, which bends their trajectories close to the Sun. Liouville’s Theorem provides a relation between the phase space distribution at 1 au relative to their distribution in the VLISM. Lee et al. (2012, 2015) used this theorem and assumption that the ionization rate inside the heliosphere decreases inversely proportional to the squared distance from the Sun to derive analytical formulae describing the IBEX-Lo observations. The restriction of the IBEX observations to a narrow range in ecliptic longitude constrains the resulting ISN flow parameters, such as velocity and temperature, to a tube in the 4D parameter space (the so-called parameter tube) with larger uncertainties along the tube (Möbius et al. 2012; McComas et al. 2012; Schwadron et al. 2015a). The IBEX observations thus result in a significant correlation of parameters describing the pristine ISN He. Since the gravitational deflection depends on the initial speed of helium atoms, the peak position provides only a correlation between the pristine flow direction and speed. The graphical representation of the uncertainty of the two parameters thus resembles a long tube (see e.g. Fig. 13) rather than a narrow ellipse. Similarly, temperature and speed are correlated because the angular extent of the observed signal represents the Mach cone angle.

Ionization of He atoms in the heliosphere modulates the ISN He populations and needs to be accounted for in the analysis of the IBEX-Lo data. Helium atoms are predominantly ionized by extreme ultraviolet radiation, but electron impact and charge exchange with solar wind He ions also contribute to this process (Ruciński et al. 1996; Bzowski et al. 2019). Ionization rates evolve significantly over the solar cycle, and electron impact ionization depends on the evolution of the electron distribution function with distance from the Sun (Bzowski et al. 2013). The time evolution of the ionization rates is crucial because the flight time of ISN He atoms from the pristine VLISM to 1 au ranges from $\sim30$ years for the primary population to $\sim200$ years for the secondary population (Bzowski and Kubiak 2020). Sokół et al. (2019c, 2020) developed a model of the ionization rates that extends over many solar cycles, using a combination of multiple observational techniques including their correlation with solar activity proxies (also see Sokół et al. 2022, Sect. 8.1, about ionization rates). These ionization models were applied in the Warsaw Test Particle Model (WTPM), which numerically tracks the atom trajectories in the heliosphere (Bzowski et al. 2012, 2015; Sokół et al. 2015). The 12 years of IBEX observations give the best-fit inflow direction $(\lambda ,\beta )=(255.59^{\circ}\pm 0.23^{\circ}, \, 5.14^{\circ}\pm 0.08^{\circ})$ in ecliptic coordinates, speed $v_{\text{HP}}=25.86 \pm 0.21~\mbox{km}\,\text{s}^{-1}$, and temperature $T_{\text{HP}}=7450\pm 140~\text{K}$ (Swaczyna et al. 2022).

Charge exchange collisions of He atoms with ions are accounted for in the total ionization rates, but elastic collisions may also influence the velocities of interstellar atoms as they propagate from the pristine VLISM to 1 au. Collisions with the solar wind ions have been considered a source of heating of interstellar atoms for a few decades (Wallis 1975; Fahr 1978; Kunc et al. 1983; Fahr et al. 1986; Chassefière et al. 1986; Gruntman 1986). Gruntman (2013, 2018) estimated that this heating in the supersonic solar wind amounts to $\sim200~\text{K}$, mostly due to collisions with solar wind protons. However, elastic collisions in the plasma region outside the heliopause have rarely been considered a source of modulation of interstellar He atoms (Chassefière and Bertaux 1987). Recently, Swaczyna et al. (2021b) demonstrated that these collisions slow down the primary ISN He by $\sim0.45~\text{km}\,\text{s}^{-1}$ and increase its temperature by $\sim1100~\text{K}$ at the heliopause compared to the properties in the pristine VLISM. The pristine VLISM speed and temperature accounting for these collisions and gravitational attraction beyond the heliopause is $v_{\text{VLISM}}=25.9~\mbox{km}\,\text{s}^{-1}$, and temperature $T_{\text{VLISM}}=6150~\text{K}$ Swaczyna et al. (2022).

### Interstellar Hydrogen

**Fig. 9:**
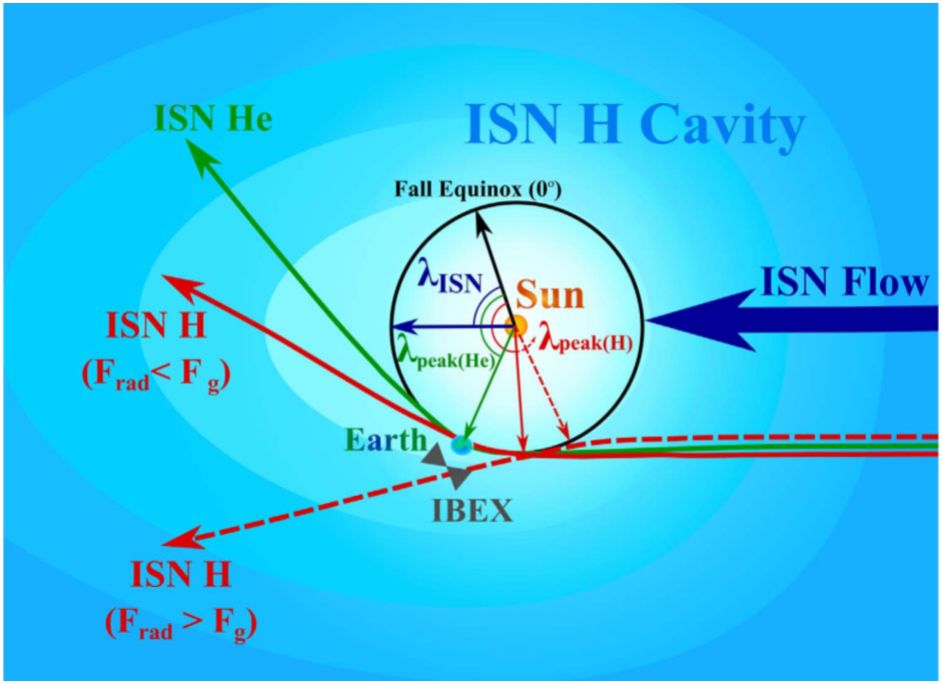
Sketch of the inflow of interstellar neutral medium. Outside the heliopause, hydrogen would be the dominant species, but close to the Sun where IBEX measures neutral atoms, the ISN H is depleted compared to the ISN He (hydrogen cavity depicted as light blue blob). Figure taken from Rahmanifard et al. (2019)

Although hydrogen is the dominant species in the unperturbed interstellar medium (H/He density $\sim 10$, Gloeckler and Geiss 2001; Müller and Zank 2004), detecting and interpreting ISN H signals in the inner solar system is challenging. In contrast to the ISN He atoms (see previous section), ISN H atoms are strongly affected by three different processes on their way toward the Sun: A substantial fraction of the primordial ISN H atoms are already filtered before reaching the heliopause. Most of the remaining ISN H atoms that cross the heliopause are ionized on their journey toward the Sun by UV photons or charge exchange with solar wind ions. In addition, solar radiation pressure largely compensates (at high solar activity even over-compensates) for the gravitational attraction of the Sun. The latter two processes cause the so-called hydrogen cavity close to the Sun (light blue oval in Fig. 9).

An important parameter to quantify these competing effects is $\mu _{0}$, the ratio of the solar radiation pressure and the solar gravitation. Depending on solar activity (and thus on solar cycle), this ratio can be smaller or larger than unity (Schwadron et al. 2013; Kowalska-Leszczynska et al. 2018; Rahmanifard et al. 2019; Katushkina et al. 2021), which results in very different trajectories of ISN H (red arrows in Fig. 9). No hydrogen focusing cone forms behind the Sun. This situation is markedly different from ISN He trajectories because radiation pressure is negligible for neutral He atoms. The ISN H signal is expected to move towards larger ecliptic longitudes (later IBEX orbits) and to become weaker during high solar activity. As a consequence, ISN H is more difficult to observe in the inner heliosphere and interpretation of the data requires more detailed knowledge about the solar activity and solar wind characteristics throughout the heliosphere compared with ISN He observations.

From the observational point of view, detection of ISN H is further complicated by the low energies of ISN H atoms at 1 au (typically $26+30 = 56~\text{km}\,\text{s}^{-1}$, i.e., $\simeq 20~\mbox{eV}$ for ram observations and well below the typical low-energy limit for ENA detectors around 10 eV for anti-ram observations). Moreover, this weak and low-energetic ISN H signal must be discerned against the much stronger ISN He signal (part of which reaches the inner solar system as a spatially extended secondary component with lower energies Kubiak et al. 2014) and other backgrounds in a neutral atom detector.

**Fig. 10:**
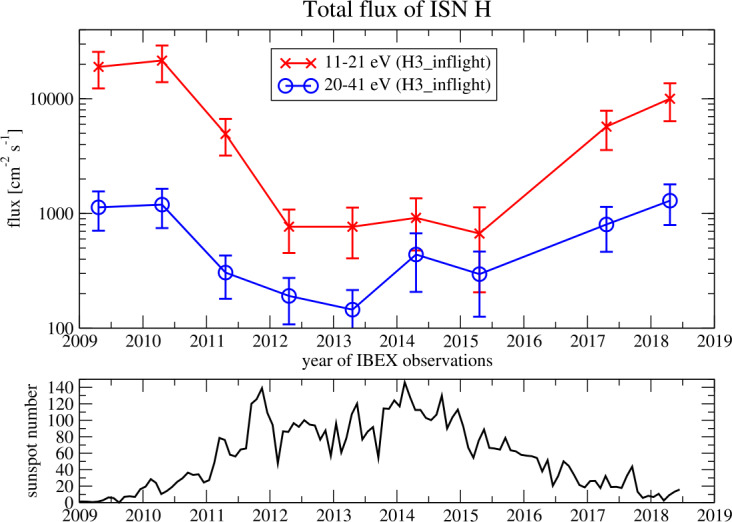
Time series of ISN H flux (top) observed with IBEX-Lo and solar activity (bottom) from 2009 to 2018. The top panel shows the total flux of ISN H in units of $\text{cm}^{-2}\,\text{s}^{-1}$ between 11 and 21 eV (red line) and between 20 and 41 eV (blue line). The data points of 2016 were omitted because ISN He observations were prioritised that year. The bottom panel shows the sunspot number as a proxy for solar activity (SILSO 2008). Figure taken from Galli et al. (2019b)

So far, IBEX-Lo has been the only instrument to ever detect ISN H atoms in-situ. The complete ISN H data obtained with IBEX-Lo from 2009–2018 were presented by Galli et al. (2019b), completing the precursor study by Saul et al. (2013). Covering almost a full solar cycle, Galli et al. (2019b) confirmed the basic theoretical expectations based on the first 4 years of IBEX-Lo observations that both the intensity and the peak longitude of observed ISN H change with solar cycle. Figure 10 illustrates this development: during high solar activity in 2013–2016, the ISN H signal could hardly be distinguished from the background. Rigorous analysis of the individual counts measured with IBEX-Lo from 2009–2011 during low solar activity in energy bins from 10 eV to 100 eV (Rodríguez Moreno et al. 2013, 2014) also allowed to identify likely interstellar D atoms. This in-situ method constrained the D/H ratio in the VLISM to $\text{D/H} = (1.6 \pm 1.0)\times 10^{-5}$, consistent with the D/H ratio of $(1.6 \pm 0.4)\times 10^{-5}$ inferred by Hubble spectroscopy for a line of sight measurement of the Local Interstellar Cloud.

**Fig. 11:**
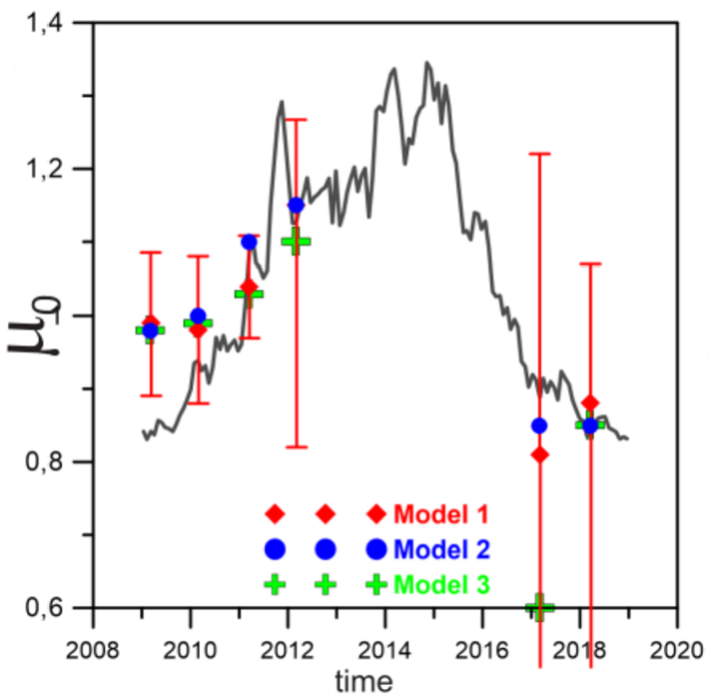
Ratio of solar radiation forces to gravity acting on ISN H ($\mu _{0}$) as a function of time for IBEX-Lo data-based values (colored symbols) versus model predictions (black line). Figure taken from Katushkina et al. (2021)

The ISN H data acquired with IBEX-Lo serve as observational constraint both for the ISN H and for Sun-related loss processes that affect ISN H in the heliosphere (Kowalska-Leszczynska et al. 2018, 2020). For instance, Rahmanifard et al. (2019) used the peak longitude of the observed ISN H signal to investigate how radiation pressure shifts the peak longitude of the ISN H inflow back and forth over the solar cycle. Rahmanifard et al. (2019), Katushkina et al. (2021) found discrepancies between the $\mu _{0}$ derived from peak longitude position and the modeled $\mu _{0}$ for individual years, albeit observations and models appear to agree when averaged over the solar cycle (see Fig. 11). The discrepancy of observed versus modeled $\mu _{0}$ “might be caused by the incorrect separation of the measured fluxes between energy channels in the data, or by some additional physical factors that are omitted in the model” (Katushkina et al. 2021). The wording “energy channels” relates to the fact that the observed energy of the ISN H (derived from the ratio of blue versus red curves in Fig. 10) is lower than predicted with models (Katushkina et al. 2015; Rahmanifard et al. 2019). More efforts both from the model and the data analysis side are required to reconcile these discrepancies and to prepare for the future ISN H measurements with IMAP-Lo (McComas et al. 2018a).

### Interstellar Oxygen and Other Heavy Species

After H and He, O is the next ISN species in abundance, followed by Ne and N. Their abundance ratios in the local interstellar medium became accessible first through measurements of PUIs (Gloeckler and Geiss 2001) and ACRs (Cummings et al. 2002). The latter are several steps further removed from the ISN source than PUIs due to further acceleration and transport of the ions. The IBEX-Lo sensor was specifically designed to also observe the anticipated ISN O flow (Möbius et al. 2009a). The first IBEX observations of the ISN flow included He, H, and O, along with the first indication of a secondary O component (Möbius et al. 2009b). These results were consistent with the same temperature for all observed ISN species and the general ISN flow direction found previously (Gloeckler et al. 2004; Möbius et al. 2004; Vallerga et al. 2004; Witte 2004).

**Fig. 12:**
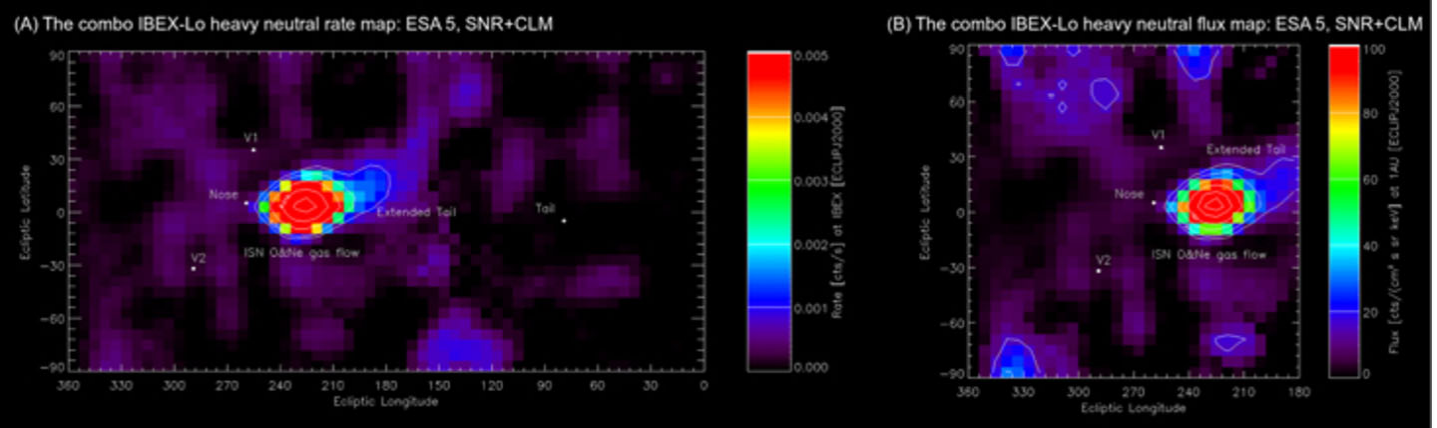
Combined IBEX-Lo sky map of heavy neutral atoms after subtracting the average background count rate in energy channel 5 in a rectangular projection with contour lines. Left: The upper confidence limits (CL = 84.13%) Right panel: The upper limits of heavy neutral fluxes in the solar inertial frame in energy channel 5, which is converted from the left panel. Adapted from Park et al. (2015)

The first complete sky maps of interstellar O & Ne atom fluxes over several years were presented by Park et al. (2014, 2015). The sky map of count rates from O and Ne in ecliptic coordinates is shown in Fig. 12. A qualitative analysis of these data showed that, along with primary interstellar oxygen atoms that directly penetrate the heliosphere from the interstellar medium, a secondary component was also measured, which is formed in the vicinity of the heliopause due to the charge exchange of interstellar O ions with H atoms ($\text{O}^{+} + \text{H} \to \text{O} + \text{H}^{+}$). The existence of a secondary oxygen population was theoretically predicted by Izmodenov et al. (1997). Filtration of interstellar oxygen at the heliospheric interface was also studied by Izmodenov et al. (1999, 2004) and Izmodenov (2007). Park et al. (2019) performed the characterization of the secondary ISN O population based on the IBEX-Lo data and estimated its velocity and temperature at the heliospheric boundary.

**Fig. 13:**
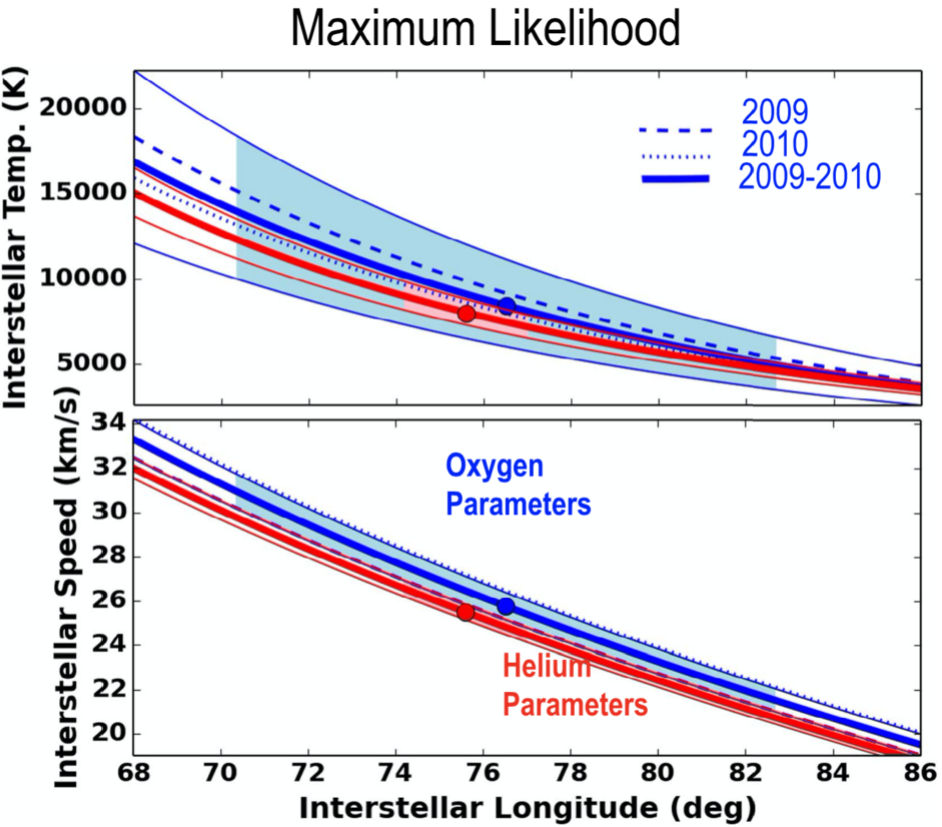
ISN O (blue) temperature (top) and speed (bottom) as a function of inflow longitude in comparison with the ISN He (red) parameters. The ISN O parameters were derived with a maximum likelihood fit to a model of the ISN flow assuming a convected Maxwellian outside the heliosphere. The functional dependences reflect the ISN parameter tube obtained with IBEX due to its limited observation range in ecliptic longitude (McComas et al. 2012). Figure taken from Schwadron et al. (2016)

A quantitative evaluation of the ISN O observations with a flow model through the heliosphere confirmed that the ISN O flow shows within uncertainties the same interstellar temperature as for He, as shown in Fig. 13 (upper panel). The O flow vector appears slightly faster and deflected relative to the ISN He flow (Fig. 13, lower panel) away from the center of the IBEX Ribbon, thought to be the direction of the interstellar magnetic field Schwadron et al. (2016). ISN O, ISN He, (mixed) ISN H, secondary He, secondary O, and Ribbon center are almost co-planar with the H deflection plane or $\mathbf{v}_{\text{{ISN}}} \times \mathbf{B}_{\text{{ISM}}}$-plane (Bzowski et al. 2019). Observing the major ISN species thus provides information about the interstellar magnetic field, the deflected plasma flow, and about its impact on the heliopause.

**Fig. 14:**
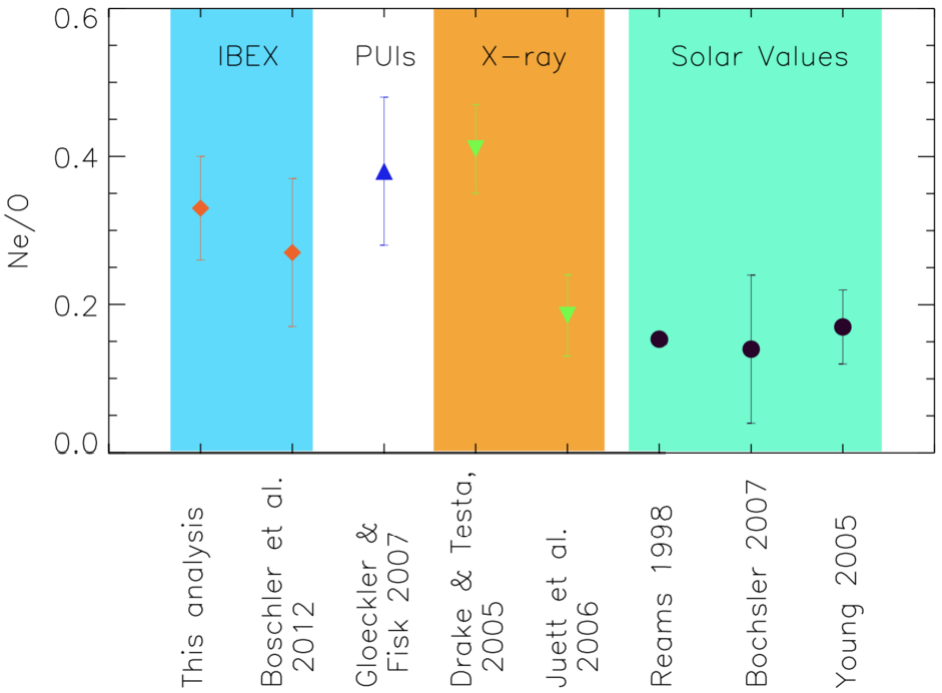
Ne/O abundance ratios in the local interstellar gas and at the Sun. The red diamonds are the results of the IBEX-Lo observations, the blue upward triangle is the value inferred by the PUIs observations, and the green downward triangles represent the interstellar abundance ratios from X-ray observations. The solar abundance values are shown as black circles. Figure taken from Park et al. (2014)

A detailed evaluation of the time-of-flight spectra of the heavy ISN component showed a substantial contribution of Ne based on its sputter products from the IBEX-Lo conversion surface, C and O. Like He, Ne does not produce stable negative ions for the analysis in IBEX-Lo. Based on the observed contribution of C in the TOF spectra, a first estimation of the Ne/O ratio at 1 AU and, drawing on modeling, in the solar neighborhood was possible (Bochsler et al. 2012). The more detailed quantitative analysis of the ISN O and Ne fluxes (Park et al. 2014, 2015) refined these results and confirmed the findings by Bochsler et al. (2012) that the Ne/O ratio in the local interstellar medium is likely somewhat higher than that in the solar system, in agreement with earlier findings based on PUI observations (Gloeckler and Fisk 2007). Figure 14 shows the ISN observations of Ne and O in context with the PUI observations and various analyses of the solar abundance ratios. Also shown are the results of two X-ray measurements of the Ne and O abundances in the interstellar medium, with one of them close to the ISN values (Drake and Testa 2005) and one of them close to the solar abundance ratio (Juett et al. 2006) (also see discussion of the Ne/O ratio measurements in Sokół et al. 2022, Sect. 8.2).

Like the ISN He observations, the O and Ne measurements suffer from the limited observation range with IBEX in ecliptic longitude. In addition, the counting statistics are low, and O and Ne observations have not been possible after the reduction in post-acceleration voltage for the IBEX-Lo instrument in 2012. The planned observations with IMAP-Lo will feature a larger geometric factor, lower background, and the capability to follow the direction of the ISN flow along the orbit around the Sun with the sensor on a pivot platform (McComas et al. 2018a). In this way, Ne and O will be better separated, and their angular distribution is obtained separately at different ecliptic longitudes. In addition, the systematic uncertainties injected by the substantial ionization loss of these species on their way to 1 AU can be largely eliminated with the observations at various ecliptic longitudes (Sokół et al. 2019b).

## Implications of Neutral Atoms Observations for the Heliosphere and Its Local Neighbourhood

In this section we review implications of the observations presented in Sect. 3 for the heliosphere and the VLISM. We explain how plasma and heliosphere characteristics can be directly inferred from ENA and ISN observations and address some of the limitations of this approach.

**Fig. 15:**
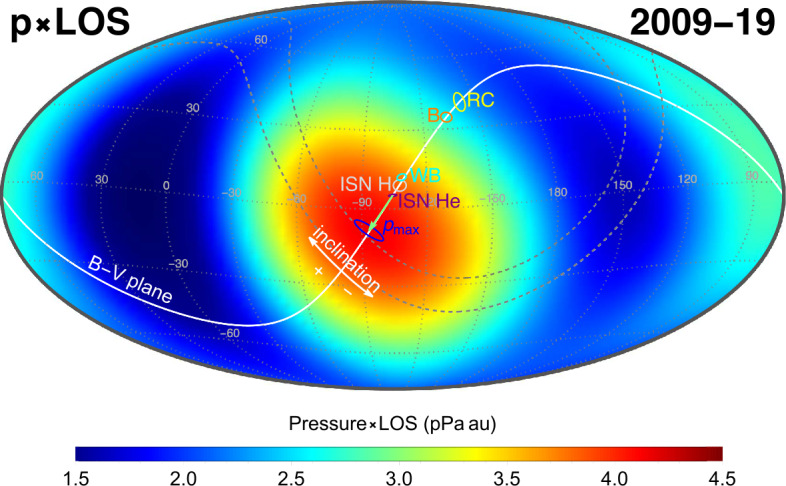
Pressure times line-of-sight (in units of pPa au) derived from the ENA intensities measured with IBEX-Hi for the entire sky. Figure taken from Swaczyna et al. (2021b)

ENA measurements of the GDF and the Ribbon with IBEX and similar space missions inform us on proton distributions inside and outside the heliosphere, combined with neutral hydrogen densities along the ENA line of sight (see Equation (1)). A quantity that can be calculated directly from the observed ENA intensity $j_{\text{{ENA}}}$ is the product of the plasma pressure of the source region times its thickness. This approach was used to derive the stationary pressure times line-of-sight maps shown in Figs. 6 and 15. Relations between the plasma ram pressure, internal pressure, and stationary pressures are discussed in Schwadron et al. (2018).

Through these relations, ENA observations put constraints on heliosphere models, but models or additional (non-ENA) observations are required to disentangle the plasma pressure and the thickness of the plasma region. Moreover, this approach implies that most or all of the observed ENAs originate from one contingent plasma source region and that the plasma bulk flow speed is known.

On its own, the observed ENA intensity cannot be attributed to different ion populations and different source regions inside and outside the heliosphere. One has to combine ENA observations with other observational constraints (keep in mind that in-situ plasma measurements in the heliosheath exist so far only for the Voyager 2 trajectory Richardson et al. 2022), rely on model results, or identify temporal variations in the ENA intensity and link these to a travel time of the parent ions and the newly created ENAs. The latter is usually done via the energy-dependent trace-back time $t_{b}(E)$ as a function of the ENA energy or ENA speed $v_{\text{{ENA}}}$. In the formulation by Zirnstein et al. (2018b), Reisenfeld et al. (2021),

2 $$ t_{b}(E) = \frac{d_{\text{{TS}}}}{v_{\text{{SW}}}} + \frac{3}{2} \frac{l_{\text{{HS}}}}{v_{ms}} + \frac{d_{TS}+l_{\text{{HS}}}/2}{v_{\text{{ENA}}}(E)}, $$

where $d_{\text{{TS}}}$ is the distance from the instrument to the termination shock, $v_{\text{{SW}}}$ is the solar wind speed traveling outwards, $l_{\text{{HS}}}$ is the a priori unknown distance through the heliosheath, and $v_{ms}$ is the magnetosonic wave speed in the heliosheath.

Assuming that all GDF ENAs originate in the heliosheath, the measured ENA intensities can be transformed into the plasma pressure in the heliosheath, which must be balanced by the plasma pressure at the heliopause. The pressure times line-of-sight map deduced from the measured GDF (shown in Fig. 15) shows that the maximum, averaged over the full solar cycle, lies on the B-V plane. The GDF ENA intensity also provides a thickness measurement for the heliosheath when Voyager 1 and 2 in-situ ion flux measurements are used as ground truth (Dialynas et al. 2022), leaving the integration length or the heliosheath thickness over the ENA fluxes as the remaining unknown. Another approach to measure the size of the heliosheath based on ENA intensities makes use of their trace-back time (Equation (2)): Using temporal pressure variations of the solar wind, the distance of the heliopause can be determined into various directions, in particular toward the heliographic poles (Reisenfeld et al. 2021).

While we seem to approach consensus about termination shock distances (Richardson et al. 2022) and heliosheath dimensions toward the nose and poles of the heliosphere with the concepts of plasma pressure and trace-back time (Reisenfeld et al. 2021), a long-standing debate within the scientific community surrounds the shape and dimension of the heliosheath in the downwind hemisphere. The ability for IBEX to place constraints on the shape of the heliosphere, heliopause is limited given the depletion of PUIs with energy. The length in which there are sufficient PUIs is called “cooling length” and it varies with energy (and assumed speeds in the heliosheath). For ENA energies from about 0.7 keV to 4 keV, the cooling length ranges from approximately 100 to 130 au (Schwadron et al. 2014b) according to:

3 $$ l_{c} = \frac {u_{R}}{n_{H} \, \sigma _{p,H} \, v_{\text{{ENA}}}}. $$

The cooling length gives an estimate of how far one can see down the heliotail with ENA maps, but ENA maps predicted for different heliosphere models or plasma parameters are required to assess the sensitivity of models and observations. Kornbleuth et al. (2021) did a comparison of two MHD models with two different shapes: the BU model (based on Opher et al. 2015) with a croissant-like shape and the Moscow model (based on Izmodenov and Alexashov 2015) with a long, comet-like shape. The BU and Moscow models were run with identical boundary conditions, and ENA fluxes were modeled from the two solutions to compare how the two models with two different shapes manifested in ENA maps. The two plasma solutions were identical until 400 au (Kornbleuth et al. 2021), showing confinement by the solar magnetic field in two jets. Beyond 400 au down the heliotail, the two solutions differ: in the BU solution the ISM flows in between the two jets while in the Moscow solution the two jets are embedded within a comet-like tail. The ENA maps produced from these two distinct solutions in the IBEX high energies showed qualitative and quantitative agreement in ENA maps. Therefore, these results indicate that IBEX-Hi observations are unable to reveal the shape of the heliotail and heliosphere. A similar comparison in IBEX-Lo remains to be done since the cooling length becomes larger at energies below 1 keV (Galli et al. 2019a). However, at these energies, the signal-to-noise ratio and background sources become more challenging (compare left and right panels in Fig. 2).

For an extended debate on the global shape of the heliosphere, the reader is referred to Sect. 8 in the model review paper by Kleimann et al. (2022). Another outstanding heliophysics question, namely if the heliopause is surrounded by a bow shock or a bow wave or a rarefaction region, is discussed in the accompanying paper by Mostafavi et al. (2022).

Two important properties of the VLISM and its influence on the heliosphere are the flow vector of neutrals $\mathbf{v}_{\text{{ISN}}}$ and the interstellar magnetic field $\mathbf{B}_{\text{{ISM}}}$. Both can be constrained from ENA and ISN observations, as shown in the previous sections: The flow vector can be derived from neutral gas observations with IBEX at 1 AU, and the direction of $\mathbf{B}_{\text{{ISM}}}$ controls the location of the Ribbon in the sky, which also provides strong constraints on its magnitude.

**Fig. 16:**
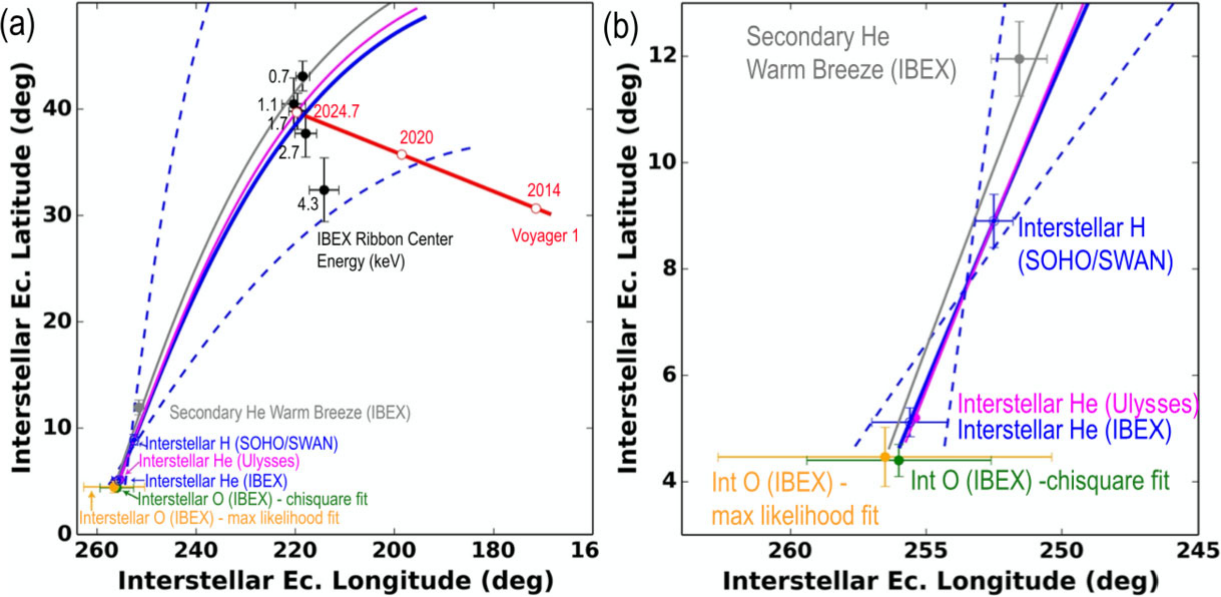
Direction of ISN H, He, and O inflow in ecliptic longitude and latitude, along with the inflow of secondary He and the Ribbon center energies (left panel). Also shown are the directions of the ISMF as observed by Voyager 1 in 2014 and its projected drift for quiet interstellar conditions (red line) and the fits indicating the $\mathbf{v}_{\text{{ISN}}} \times \mathbf{B}_{ \text{{ISM}}}$-plane based on the various ISN flow components. The blue curve shows the $\mathbf{v}_{\text{{ISN}}} \times \mathbf{B}_{ \text{{ISM}}}$-plane based on measurements from SOHO/SWAN and IBEX with the dashed blue curves indicating the uncertainty limits. The $\mathbf{v}_{\text{{ISN}}} \times \mathbf{B}_{ \text{{ISM}}}$-plane based on measurements from SOHO/SWAN and Ulysses is shown as purple curve. The right panel shows a blow-up from the left panel centering on the ISN measurements. Figure taken from Schwadron et al. (2016)

Furthermore, the offset of the Ribbon from an expected nominal position at $90^{\circ}$ from the pristine field direction (if the Ribbon source were located in the pristine ISM) provides insight on the draping of the interstellar magnetic field around the heliopause. The secondary interstellar neutral atom distribution, which arises from charge exchange between interstellar ions and ISN gas outside the heliopause, provides insight into the interstellar plasma flow pattern as it is diverted around the heliopause and drags along the interstellar magnetic field. This interaction leads to a heliosphere and flow pattern that is organized by the $\mathbf{v}_{\text{{ISN}}} \times \mathbf{B}_{ \text{{ISM}}}$-plane, as illustrated in Fig. 16. The most common interpretation of the IBEX Ribbon as an ENA source from outside the heliopause (see Sect. 3) is an independent confirmation of the insight provided by the primary and secondary ISN populations.

The observed inflow of interstellar heavy atoms also constrains heliosphere models provided they take into account both the filtration of primary and the production of secondary interstellar oxygen in the boundary region of the heliosphere and they explicitly calculate the motion of interstellar atoms inside the heliosphere. That calculation must take into account photoionization, charge exchange with the solar wind protons, and solar gravity. Accordingly, Baliukin et al. (2017) modeled the trajectories of ISN oxygen and neon through the heliosphere based on the three-dimensional kinetic-MHD model by Izmodenov and Alexashov (2015) and compared the results with the IBEX observations. Baliukin et al. (2017) found agreement between the modeled and the observed count rates within the 35% IBEX-Lo instrument uncertainty. The model produced a similar extended tail of secondary ISN oxygen as observed, albeit less intense, but within the observational error bars.

One of the key parameters that influences the direction and intensity of the secondary oxygen component is the interstellar magnetic field. The influence of the interstellar magnetic field leads to an asymmetry in the heliosphere and deflection of the plasma flow in the vicinity of the heliopause. As a result, neutral oxygen atoms that originated from charge-exchange of O ions in this region (secondary component) show a deviation in their average velocity from the direction of the interstellar wind. Consequently, the configuration of the interstellar magnetic field near the heliopause affects the spatial distribution of atoms inside the heliosphere.

To illustrate this point, we calculated for this review the velocity distribution function of O atoms using the global model of solar wind-VLISM interaction (Baliukin et al. 2017) for two different ISMF configurations: 1) $B_{\mathrm{VLISM}} = 4.4~\upmu \text{G}$ and angle between $\mathbf{B}_{\mathrm{VLISM}}$ and $\mathbf{v}_{\mathrm{VLISM}} = 20^{\circ}$ (Izmodenov and Alexashov 2015); and 2) $B_{\mathrm{VLISM}} = 3.75~\upmu \text{G}$ and angle between $\mathbf{B}_{\mathrm{VLISM}}$ and $\mathbf{v}_{\mathrm{VLISM}} = 60^{\circ}$ (reproducing the Voyager 1 and 2 observations Izmodenov and Alexashov 2020).

**Fig. 17:**
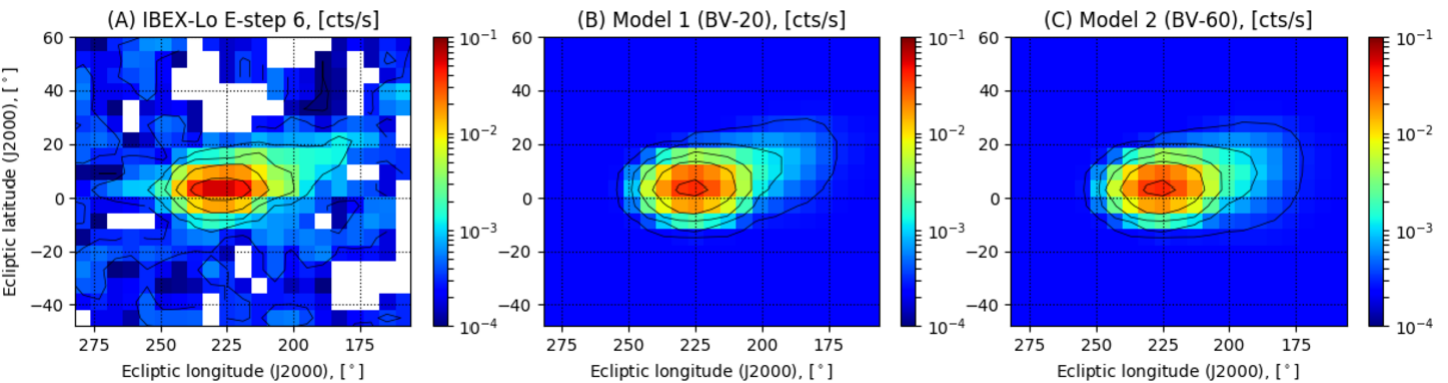
(**A**) Skymap (in ecliptic coordinates) of count rates due to O & Ne atoms as observed with IBEX-Lo (Park et al. 2015); (**B**) skymap according to model predictions (Baliukin et al. 2017) using configuration 1; (**C**) skymap according to model predictions (Baliukin et al. 2017) using configuration 2

Figure 17 shows the skymap of O & Ne atom fluxes in ecliptic coordinates according to the IBEX-Lo data of energy step 6 (panel A) and the model predictions for the two different ISMF configurations (panel B: configuration 1, panel C: configuration 2). A similar structure is visible in all maps: an extended tail toward lower longitude and higher latitude (secondary component) adjacent to the region of maximal fluxes (primary inflow of neutral oxygen and neon, shown in red in the figures). Model 2 predicts the higher (by $\approx50\%$) amount of secondary hydrogen atoms at the heliopause, which is closer to IBEX-Lo data compared to Model 1. However, the differences between the modeled skymaps of O & Ne atoms are almost negligible (cf. panels B and C). These results suggest a weak dependence of the O & Ne tail on the ISMF configuration and they illustrate that the IBEX observation geometry together with the IBEX-Lo instrument characteristics limit the quantitative diagnostics of the magnetic field at the heliosphere boundary.

## Conclusions and Outlook

This review has shown in how many ways ENA images and the direct sampling of several species of the ISN flow contribute to our understanding of the morphology of the heliosphere, the kinetic state and the composition of the surrounding interstellar medium, and the interaction between the two domains. The combination of interstellar flow, thermal pressure, and magnetic pressure set the outer boundary conditions for and shape the heliosphere. The pressure distribution at the heliopause is balanced by the pressure in the heliosheath, which is seen reflected in the GDF of ENAs. Combining the observations of the ISN flow, the Ribbon, secondary neutrals, and the GDF, along with the Voyager crossings of the termination shock and the heliopause, provides multiple constraints for the global heliospheric models. In this way, the heliosphere and its interaction with the local interstellar medium can be described with ever growing sophistication.

While there have been numerous advances in global models, they are far from being able to capture all critical aspects of the heliosphere such as the size, shape, flows in the heliosheath, and they fail so far to explain some major observations. Models do not integrate the influence of suprathermal particles, reconnection, turbulence phenomena, and other microprocesses. The transport of Cosmic Rays and the evolution of PUIs require kinetic transport models that are coupled self-consistently to a global MHD model. Development of such a model is currently being undertaken by the SHIELD NASA Drive Center.^1^ For ENA modeling it is particularly critical to include PUIs modeled kinetically self-consistently in the heliosphere.

The follow-up mission on IBEX, the Interstellar Mapping and Acceleration Probe (IMAP) is scheduled for launch in 2025 (McComas et al. 2018a). It will include, among other in-situ and remote sensing instruments, improved versions of IBEX-Lo (IMAP-Lo) and IBEX-Hi (IMAP-Hi), as well as IMAP-Ultra to measure ENAs up to 300 keV. The vantage point at the L1 point instead of an Earth orbit and mounting IMAP-Lo on a pivot platforms will improve the duty cycle and the signal-to-noise ratios for many ENA observations and ISN species by an order of magnitude at least compared with IBEX. From this review, we can derive the most important wishes for IMAP for each of the subtopics discussed:

- GDF: Better statistics and lower background in annual sky maps, in particular at energies below 0.5 keV, to attribute the ENAs to the different plasma sources inside and possibly outside the heliopause, which will help us better constrain the physics and the dimensions of the heliosphere.
- Ribbon: Higher spatial resolution around solar wind energy to verify creation mechanisms, locate Ribbon region, and study temporal effects.
- For all heliospheric ENAs: wider and overlapping energy ranges to verify the energy spectrum (slopes and potential knees). This will also help to clarify the relation between the IBEX Ribbon and the INCA belt.
- ISN H: expand observation time and spread per year and re-check energy of incoming atoms. Determine a more precise ISN D/H ratio.
- ISN He: break the inflow parameter degeneracy by observing ISN He over a wide range of longitudes that result in multiple parameter tubes with different orientations. The intersection of the parameter tubes will unambiguously determine the real inflow parameters.
- ISN heavy atoms: detect the inflow also from anti-ram direction, capture the full distribution of the secondary inflow, and separate between Ne and O using their vastly different ionization rates at different longitudes of observation.

## Footnotes

1 https://sites.bu.edu/shield-drive/.
